# Molecular dynamics approach to identification of new OGG1 cancer-associated somatic variants with impaired activity

**DOI:** 10.1074/jbc.RA120.014455

**Published:** 2021-01-07

**Authors:** Aleksandr V. Popov, Anton V. Endutkin, Darya D. Yatsenko, Anna V. Yudkina, Alexander E. Barmatov, Kristina A. Makasheva, Darya Yu. Raspopova, Evgeniia A. Diatlova, Dmitry O. Zharkov

**Affiliations:** 1 Laboratory of Genome and Protein Engineering, SB RAS Institute of Chemical Biology and Fundamental Medicine, Novosibirsk, Russia; 2 Department of Natural Sciences, Novosibirsk State University, Novosibirsk, Russia

**Keywords:** DNA damage, DNA repair, DNA glycosylases, OGG1, genetic polymorphism, personalized medicine, protein function prediction, structure–function, molecular dynamics, AP, apurinic/apyrimidinic, BER, base excision repair, COSMIC, Catalogue Of Somatic Mutations In Cancer, MD, molecular dynamics, OGG1, 8-oxoguanine DNA glycosylase, oxodG, 8-oxo-2′-deoxyguanosine, oxoG, 8-oxoguanine, PCA, Principal component analysis, THF, (3-hydroxytetrahydrofuran-2-yl)methyl phosphate

## Abstract

DNA of living cells is always exposed to damaging factors. To counteract the consequences of DNA lesions, cells have evolved several DNA repair systems, among which base excision repair is one of the most important systems. Many currently used antitumor drugs act by damaging DNA, and DNA repair often interferes with chemotherapy and radiotherapy in cancer cells. Tumors are usually extremely genetically heterogeneous, often bearing mutations in DNA repair genes. Thus, knowledge of the functionality of cancer-related variants of proteins involved in DNA damage response and repair is of great interest for personalization of cancer therapy. Although computational methods to predict the variant functionality have attracted much attention, at present, they are mostly based on sequence conservation and make little use of modern capabilities in computational analysis of 3D protein structures. We have used molecular dynamics (MD) to model the structures of 20 clinically observed variants of a DNA repair enzyme, 8-oxoguanine DNA glycosylase. In parallel, we have experimentally characterized the activity, thermostability, and DNA binding in a subset of these mutant proteins. Among the analyzed variants of 8-oxoguanine DNA glycosylase, three (I145M, G202C, and V267M) were significantly functionally impaired and were successfully predicted by MD. Alone or in combination with sequence-based methods, MD may be an important functional prediction tool for cancer-related protein variants of unknown significance.

DNA of living cells is always exposed to a number of damaging factors, both endogenous and environmental. This causes modification of nucleobases and formation of abasic (apurinic/apyrimidinic, or AP, sites) and DNA breaks. The accumulated DNA lesions negatively affect the genome stability, leading to gene inactivation or changes in the properties of the encoded proteins. Most DNA lesions, such as deaminated, oxidized, and alkylated bases and single-strand breaks, are fixed by the base excision repair (BER) system (1, 2). BER is initiated by the recognition of a lesion by one of the enzymes belonging to the group of DNA glycosylases, which hydrolyze the *N*-glycosidic bond between the nucleobase and C1′ of deoxyribose, forming an AP site. Another enzyme, AP endonuclease, then hydrolyzes the phosphodiester bond 5′ to the AP site. The repair cycle is finished after the incorporation of the missing deoxynucleoside monophosphate and ligation of the remaining nick (1, 2). In addition to BER, several other DNA repair systems have been described in human cells: direct reversal, nucleotide excision repair, mismatch repair, recombination repair, and so forth (1).

Many currently used antitumor drugs act by damaging DNA, causing cytotoxicity in rapidly dividing cells (3). Thus, DNA repair systems, being absolutely required for genome maintenance in normal cells, often interfere with chemotherapy and radiotherapy in cancer cells. Inhibition of DNA repair is presently considered an efficient strategy to sensitize tumors toward drugs and ionizing radiation, and recently, inhibitors of poly(ADP-ribose)polymerase, a key BER regulator (4, 5), as well as inhibitors of *O*^6^-methylguanine methyltransferase, an enzyme that repairs alkylated guanines (6, 7), have been approved for clinical use. Several inhibitors of other targets in BER and other repair systems are presently at various stages of clinical trials.

Tumors are usually extremely genetically heterogeneous because of their inherent genetic instability and fast clonal proliferation (8, 9). Among other genetic changes, cancer cells often bear mutations in DNA repair genes (10, 11). Most mutations in the protein-coding regions of cancer cell genomes are missense variants of unknown significance (12). To expand options for personalized cancer treatment, information about the functionality of variants of proteins involved in DNA damage response and repair is of great interest. Because at present it is easier to obtain genotype data than to functionally characterize variants of unknown significance, computational methods to predict the variant functionality have attracted much attention (13, 14). However, direct benchmarking of such predictions against phenotypically characterized variants of cancer-related genes with known clinical significance still produces widely divergent results, preventing the use of currently used algorithms in practical cancer treatment personalization (15). The development of new computational ways to predict the effect of mutations in protein-coding regions is therefore of utmost importance.

At present, the majority of algorithms used for the prediction of the effects of mutations on the protein functions are based on the analysis of evolutionary conservation, changes in the amino acid physicochemical properties, and the available static structures rather than on the modeling of the dynamic behavior of the protein molecule (16, 17, 18, 19, 20, 21, 22, 23, 24, 25, 26). Some modern prediction protocols use neural network and machine learning methods to integrate various kinds of information (27, 28, 29). Molecular dynamics (MD), due to its much higher computational power demands and the lack of experimentally determined 3D structures for many proteins, had seldom been used for the prediction of protein variant functionality. However, with the number of known structures growing quickly, and processor capabilities and computational algorithms improving rapidly, this approach is gaining in popularity (30, 31). For example, MD was used to predict the inhibitor binding with 12 commonly found somatic variants of BCR-ABL1 oncogenic protein kinase (32). In another recent study, structures of 79 naturally occurring nonsynonymous SNPs of human triose phosphate isomerase were analyzed, and some were found to destabilize the protein fold (33). There is little doubt that MD will soon become a powerful approach for the prediction of mutation effects on the protein functionality either on its own or as an important part of combined procedures integrating structural and evolutionary information.

In this study, we have developed a semiautomated pipeline to model the structures of somatic mutant variants of proteins found in clinical samples of human tumors and predict the effects of the mutations. As a proof of principle, we have applied the procedure to mutants of 8-oxoguanine DNA glycosylase (OGG1). DNA glycosylases, as many other BER enzymes, modulate the resistance of cancer cells to chemotherapy and radiotherapy. OGG1 initiates the repair of oxidized purines, mostly 8-oxoguanine (oxoG) and formamidopyrimidines. In cell cultures, OGG1 significantly attenuates the toxicity of bleomycin (34), thiotepa (35, 36), carmustine (36, 37), mafosfamide (36), cisplatin, and oxaliplatin (38). Thus, somatic mutations in the *OGG1* gene in tumor cells could affect their sensitivity to therapeutic interventions. Using the computational pipeline, we have identified three novel clinical variants of OGG1 with impaired activity.

## Results

### Predicting the mutation effect in OGG1 variants

At the beginning of this study, the Catalogue Of Somatic Mutations In Cancer (COSMIC) database (39) held 39 nonsynonymous single-nucleotide mutations in the *OGG1* gene. We have used four popular software packages for mutation effect prediction from sequence conservation—SIFT (18), FATHMM (24), MutationTaster (25), and PROVEAN (22)—to analyze the functionality of these mutations. All of them produce a binary output: whether the protein function is expected to be affected or not affected in some way. Of 39 substitutions, 36 were included and three (all located in exon 8, the last exon of the human gene) were discarded because of the insufficient number of homologous sequences (Table S1). Cohen’s κ coefficient (40) was used to measure the agreement between the prediction methods (−1 ≤ κ ≤ 1; where κ = −1 for complete disagreement, κ = 1 for complete agreement, and κ = 0 for agreement expected from random coincidence). As can be seen from Table 1, the agreement between different algorithms was rather low in most cases. For a comparison, we took the gene for another human DNA glycosylase, *UNG*, and repeated the analysis for nonsynonymous single-nucleotide mutations found in the COSMIC database its coding region (44 total, all included; Table S1). Neither the *UNG* mutation set nor the combined *OGG1*/*UNG* set demonstrated better performance than the *OGG1* set (Table 1). Given the discordance in the predictions by sequence-based algorithms, we set out to apply MD for estimating the mutation effects in the *OGG1* coding sequence.

**Table 1:** Cohen’s κ for agreement between the predictions of the effect of mutations in *OGG1* and *UNG* by different algorithms

	FATHMM	MutationTaster	PROVEAN
SIFT	0.163^a^	0.385	0.780
	0.068	0.323	0.178
	0.087	0.428	0.490
FATHMM		0.192	0.289
		0.049	0.117
		0.079	0.177
MutationTaster			0.503
			0.371
			0.464

a First number is Cohen’s κ for the *OGG1* mutation set, second, for the *UNG* mutation set, third, for the combined set.

### MD of OGG1 variants

**Figure 1:**
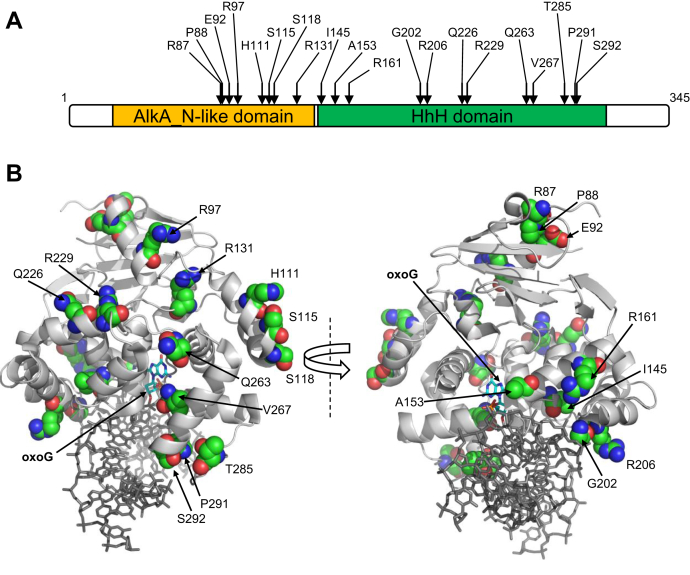
**Location of the studied mutations in the primary structure (*A*) and the tertiary structure of OGG1 (*B*).** In the primary structure, AlkA_N-like domain is homologous to the N-terminal domain of *Escherichia coli* 3-methyladenine–DNA glycosylase AlkA, and the HhH domain contains a helix–hairpin–helix motif found in many DNA glycosylases. In the tertiary structure (1EBM, (43)), the mutated residues are shown as *colored spheres* and the everted oxoG nucleotide as *colored sticks*. OGG1, 8-oxoguanine DNA glycosylase.

We have designed a computational pipeline that retrieves all missense variants of the gene of interest from the COSMIC database (or, with an appropriate setup of the connection module, from any database that can be automatically parsed), maps them on the selected protein sequence and structure, randomly selects a given number of mutants, and prepares them for MD. The script is available as an open source code from https://doi.org/10.5281/zenodo.3828057. Mapping is an important part of the procedure because many protein isoforms are known for OGG1 (41, 42), and the COSMIC database uses the longest polypeptide, the major mitochondrial isoform OGG1-2a, as a reference, whereas the vast majority of biochemical and all structural data in the literature are for the shorter major nuclear isoform OGG1-1a. Of the randomly selected 20 mutants, only one (R229Q) turned out to be studied before with respect to its biochemical properties, and for another one (R131G), a substitution of Gln for the same Arg131 residue was characterized as inactive (Table 2). The location of the mutants in the sequence and structure of OGG1 protein is shown in Figure 1. All 20 mutants and WT OGG1 were subjected to MD in explicit water.

**Table 2:** Somatic mutants of OGG1 investigated in this study

OGG1 variant	Predicted effect^a^						Properties reported in the literature
	SIFT	FATHMM	MutationTaster	PROVEAN	Combined score	Experimental	
R87K	No	No	No	No	+4		
P88L	No	No	Yes	No	+2		
E92D	No	No	Yes	No	+2	No	
R97C	Yes	No	No	Yes	0		
H111R	No	No	No	No	+4	No	
S115F	Yes	No	No	Yes	0		
S118F	Yes	No	Yes	Yes	−2	No	
R131G	Yes	Yes	Yes	Yes	−4		R131Q: small-cell lung carcinoma; inactive (86, 87)
I145M	Yes	No	Yes	Yes	−2	Yes	
A153T	No	Yes	No	No	+2		
R161W	Yes	Yes	Yes	Yes	−4	Yes/No	
G202C	Yes	Yes	Yes	Yes	−4	Yes	
R206C	Yes	Yes	Yes	Yes	−4	No	
Q226H	No	No	Yes	No	+2	No	
R229Q	No	No	No	Yes	+2		Leukemia; active but thermolabile at 37 °C; cells radiation sensitive (88, 89, 90)
Q263H	Yes	No	Yes	No	0	No	
V267M	Yes	No	Yes	Yes	−2	Yes	
T285M	No	No	No	No	+4	No	
P291Q	Yes	No	No	No	+2	No	
S292N	No	No	No	No	+4	Yes/No	

a Binary output from individual algorithms, coded as the following: Yes: SIFT “affects protein function,” FATHMM “damaging,” MutationTaster “disease causing,” PROVEAN “deleterious”; No: SIFT “tolerated,” FATHMM “tolerated,” MutationTaster “polymorphism,” PROVEAN “neutral.” Combined score: sum of the four prediction, with +1 for “No” and −1 for “Yes”; negative values indicate an effect on the protein function.

Forty ns trajectories were essentially equilibrated after 20 ns (Fig. S1), so the last 20 ns were analyzed. To extract functionally relevant differences between the mutants, we focused on the enzyme’s active site, defined as all residues in protein and DNA, at least one atom of which is within 5 Å of 8-oxo-2′-deoxyguanosine (oxodG) in the OGG1–DNA complex X-ray structure (1EBM (43)) that served as a starting point for our models. Thus defined, the active site encompasses 23 amino acid residues and three nucleotides: oxodG and its 5′ and 3′ neighbors. Of all studied mutations, only V267M affected a residue in the active site, which includes N and Cα atoms of Val267. In addition, we analyzed a narrower set of key geometric parameters for the catalytic dyad Lys249–Asp268, namely the Nζ[K249]–C1′[oxodG] and Oδ1/Oδ2[D268]–O4′[oxodG] distances and the Nζ[K249]–C1′[oxodG]–N9[oxodG] angle. These three parameters reflect correct positioning of the catalytic amine nucleophile of Lys249 and the damaged nucleotide (43, 44) and were earlier shown to be good predictors for the OGG1 activity on a limited set of substrates (oxoG:C, oxoG:A, and AP:C) and active site–obstructing mutants (45, 46).

### Trajectory analysis and clustering

**Figure 2:**
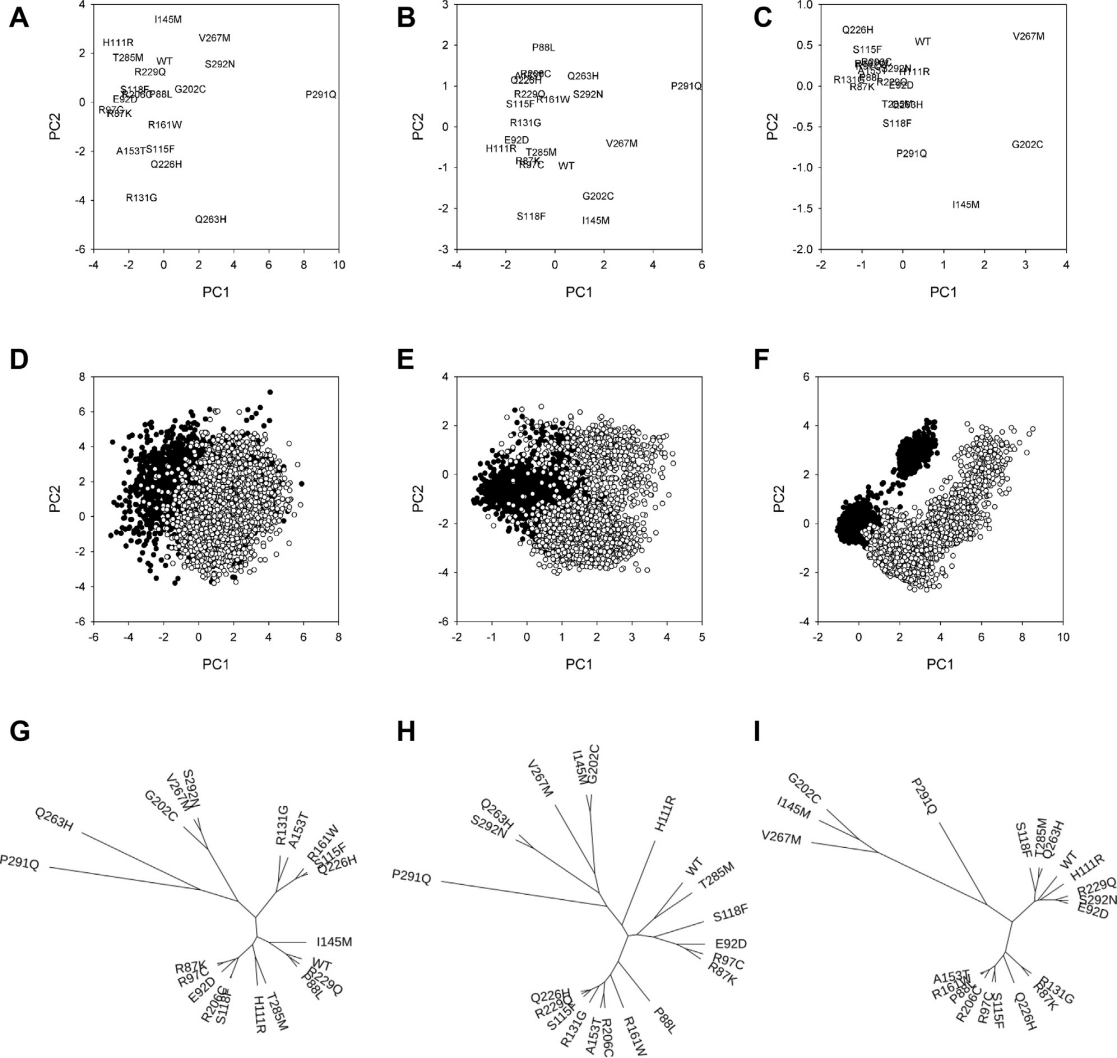
**Principal component analysis of OGG1 mutants.***A*–*C*, location of all OGG1 mutants (means of principal components 1 and 2) in the PC1 *versus* PC2 plane. *D*–*F*, all trajectory points of WT OGG1 (*black dots*) and one of the mutants, OGG1 G202C (*white dots*) in the PC1 *versus* PC2 plane. *G*–*I*, unrooted distance tree derived from the Bhattacharya similarity coefficients in the PC1 *versus* PC2 plane. The PCA was performed for the whole active site (*A*, *D*, and *G*), only the torsions within the active site whose mean deviated >36° from WT OGG1, and at least one mutant (*B*, *E*, and *H*), or for three critical geometric parameters (*C*, *F*, and *I*). OGG1, 8-oxoguanine DNA glycosylase; PCA, Principal component analysis.

Principal component analysis (PCA) is often used to reduce dimensionality of multivariate data with minimal information loss. We have first performed the PCA using the dihedral covariance matrix taking into account all torsion angles of the active-site residues for all analyzed variants. Principal components 1 and 2 for all OGG1 mutants are illustrated in Figure 2*A*. To cluster the full trajectories rather than their means (*e.g.*, as presented in Fig. 2*D*), we have calculated Bhattacharya similarity coefficients in the PC1 *versus* PC2 plane for all pairs of trajectories (see Experimental procedures) and presented the results as a tree (Fig. 2*G*). The variants formed several tight clusters; P88L and R229Q were closest to the WT enzyme, and I145M was close to this group. Q263H and P291Q lied farthest from WT OGG1 and were widely separated from all other proteins.

High dimensionality of multivariate data introduces large dispersion into principal components, lowering the ability of the PCA to separate the distributions coming from different samples. We tried to reduce the number of dimensions by including only those torsions within the active site that demonstrated large differences (>36° difference in the means) between WT OGG1 and at least one mutant (Fig. 2, *B*, *E* and *H*). The trajectories were better separated (Fig. 2*E*), and, after tree building, two clusters were evident, one containing WT OGG1 and E92D, R87K, R97C, S118F, and T285M mutants, the other consisting of P88L, S115F, R131G, A153T, R161W, R206C, Q226H, and R229Q. Of the remainder, I145M grouped together with G202C, and Q263H, with S292N.

When we repeated the PCA with the three key active site geometric parameters as the only variables, the principal component projections were considerably different (Fig. 2, *C* and *F*). Owing to a lower number of variables, the clustered structure of the population of WT OGG1 active site conformations, noted in the earlier OGG1 models (45, 46), was more evident in the first two PC dimensions than with the 5-Å active site. In Figure 2*F* and Fig. S2, the conformations of the WT OGG1 with an optimal geometry for an attack at C1′ (45, 46) crowd around the origin of coordinates in the PC1 *versus* PC2 plane. The tree grouped WT OGG1 with E92D, H111R, S118F, R229Q, Q263H, T285M, and S292N mutants, whereas R87K, P88L, R97C, S115F, R131G, A153T, R161W, R206C, and Q226H produced another cluster. I145M, G202C, and V267M formed a cluster remote from most of other mutants, whereas P291Q was an isolated branch. Comparing individual trajectories in the PC1 *versus* PC2 plane, one can notice that I145M, G202C, and V267M are very different from WT OGG1 and other mutants, while P291Q and other variants are close to the “optimal” cluster.

### Biochemical characterization of OGG1 variants

**Figure 3:**
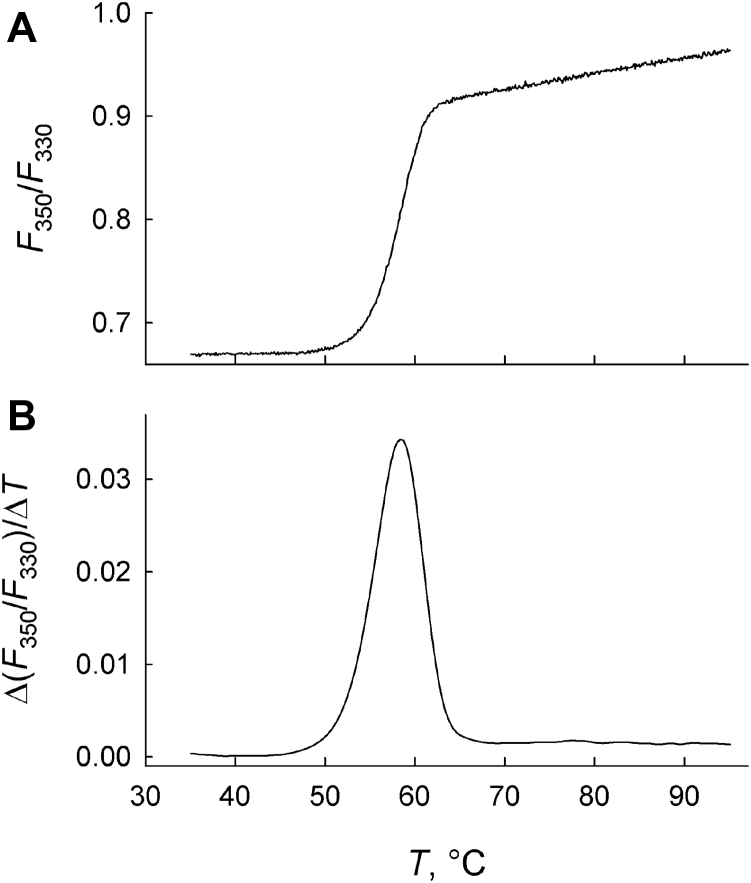
**Thermal denaturation of a representative OGG1 mutant, OGG1 H111R.***A*, Trp fluorescence (*F*_350_/*F*_330_) melting profile. *B*, differential Trp fluorescence melting profile (two averaged melting curves). OGG1, 8-oxoguanine DNA glycosylase.

Of twenty OGG1 mutants analyzed by MD, 13 plus the WT were successfully overproduced and purified (Fig. S3), whereas nine were either very poorly induced or insoluble upon induction in *Escherichia coli*. All polypeptides carried an N-terminal His_6_-tag, which, according to the known OGG1 structure (43), lies in an unstructured part remote from the active site and is unlikely to affect the protein function. The effect of the mutations on the protein stability was estimated from their Trp fluorescence melting profiles (Fig. 3). All mutants and the WT protein demonstrated a single transition point with *T*_m_ ranging between 55.9 °C and 59.4 °C, indicating no significant destabilization (Table 3). The mutations did not appreciably change the F_350_/F_330_ ratio at the physiological temperature, suggesting that all mutants have the same general conformation as WT OGG1.

**Table 3:** Thermal denaturation of OGG1 mutants

OGG1 variant	*T*_m_, °C	F_350_/F_330_ at 35 °C
WT	58.4 ± 0.1	0.67 ± 0.01
E92D	58.1 ± 0.2	0.66 ± 0.01
H111R	58.4 ± 0.1	0.67 ± 0.01
S118F	56.2 ± 0.1	0.67 ± 0.01
I145M	57.2 ± 0.1	0.67 ± 0.01
R161W	55.9 ± 0.2	0.72 ± 0.02
G202C	57.1 ± 0.1	0.67 ± 0.01
R206C	56.8 ± 0.2	0.70 ± 0.01
Q226H	58.1 ± 0.1	0.66 ± 0.01
Q263H	55.9 ± 0.1	0.66 ± 0.01
V267M	56.2 ± 0.1	0.67 ± 0.01
T285M	56.4 ± 0.1	0.69 ± 0.01
P291Q	56.2 ± 0.2	0.68 ± 0.01
S292N	59.4 ± 0.1	0.66 ± 0.01

OGG1, 8-oxoguanine DNA glycosylase.

**Figure 4:**
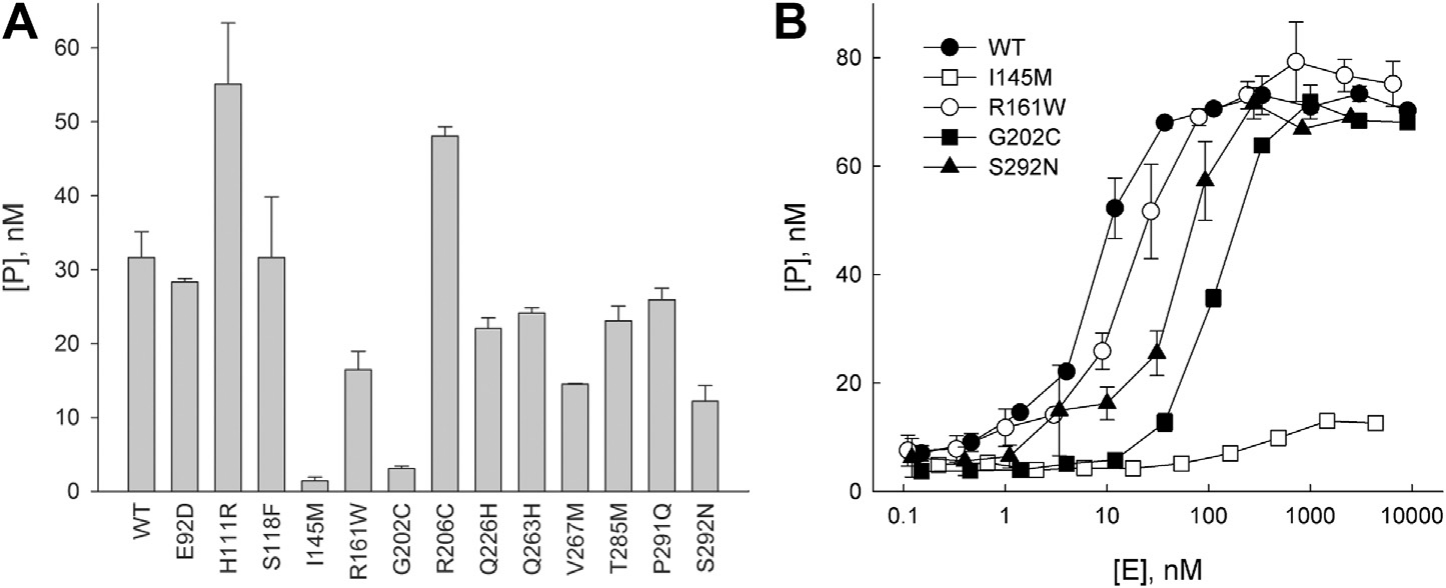
**Relative oxoG excision activity of WT and mutant OGG1 proteins.***A*, single time-point assay (mean ± SE, *n* = 3–7). *B*, dependence of cleavage substrate on the protein concentration. *Black circles*, WT OGG1; *white circles*, R161W; *squares*, I145M; *triangles*, S292N. *Symbols* indicate mean ± SE (*n* = 3). OGG1, 8-oxoguanine DNA glycosylase; oxoG, 8-oxoguanine.

The overall activity of OGG1 variants was assessed in a single time-point assay using an oligonucleotide duplex containing an oxoG:C pair, the natural substrate for this enzyme (47) (Fig. 4*A*). Two variants, I145M and G202C, displayed the activity ∼10- to 20-fold lower than the WT enzyme, whereas the cleavage by R161W, V267M, and S292N was ∼1.5- to 2-fold lower. These findings were corroborated by enzyme titration experiments, which demonstrated very low cleavage by I145M even at the highest enzyme concentrations (Fig. 4*B*). G202C activity was more than 10-fold lower, whereas R161W and S292N mutations showed a milder effect. Interestingly, some of the mutants, such as H111R and R206C, were apparently more active than WT OGG1.

To get a deeper insight into possible reasons of the variation in the activity of OGG1 mutants, we have determined the apparent rate constants *k*_2_ and *k*_3_. OGG1 is characterized by fast oxoG excision and slow dissociation from the DNA product, so *k*_2_ reflects the base excision rate, whereas *k*_3_ is a combined constant accounting for the nascent AP site cleavage and product release (48, 49). Single-turnover and burst phase experiments are used to determine *k*_2_ and *k*_3_ for OGG1 and other slow-turnover DNA glycosylases (48, 50). For comparison with the WT OGG1, we have selected four mutants with low activity (I145M, R161W, G202C, and S292N), two high-activity variants (H111R and R206C), and an apparently neutral Q263H. The results are summarized in Table 4 and Figs. S4 and S5. The low activity of I145M did not allow reliable determination of the kinetic constants. For G202C, no reliable *k*_2_ and *k*_3_ values could be obtained: although the apparent *k*_2_ was decreased at least 50-fold, and *k*_3_ was 4.5-fold lower compared with the WT enzyme, as reported in Table 4, the actual constants may be even more affected, given that at the highest enzyme concentration, the reaction with G202C did not reach the endpoint (Fig. S4). V267M was also affected, with *k*_2_ showing a ∼12-fold decrease. R161W and S292N, which demonstrated mild overall effect of the mutation (Fig. 4), were also the least affected in kinetic terms, showing only a ∼2-fold *k*_2_ decrease. Although some of the affected mutants may not fully bind the substrate at 800 nM (see below), the reported values provide a useful lower estimate of *k*_2_. In WT OGG1, H111R, R206C, Q263H, and P291Q variants, we could provide only lower estimates for *k*_2_ because the cleavage was very fast even at 15 °C, the temperature at which the experiment was performed. With this precaution, the “hyperactive” H111R and R206C showed a 2- to 2.5-fold increase in the apparent *k*_2_ with about the same decrease in *k*_3_, whereas the “neutral” Q263H had unaffected *k*_2_ and the highest *k*_3_ of all studied variants. Overall, it seems that in this particular set of variants, the activity measured in single time-point experiments better correlates with the base excision rate constant. Based on these experiments, we classified I145M, G202C, and V267M as “affected” and R161W and S292N as “possibly affected.”

**Table 4:** Kinetic parameters of OGG1 mutants

OGG1 variant	*k*_2 app_, min^−1^	*k*_3 app_, min^−1^
WT	>2.6 ± 0.4^a^	0.027 ± 0.014
H111R	>5.1 ± 0.5	0.012 ± 0.010
I145M	n/c^b^	n/c
R161W	1.6 ± 0.2	0.018 ± 0.011
G202C	0.026 ± 0.004	0.006 ± 0.005
	0.048 ± 0.012^c^	
R206C	>7.1 ± 1.9	0.013 ± 0.004
Q263H	>2.8 ± 0.3	0.084 ± 0.022
V267M	0.21 ± 0.04	0.015 ± 0.010
P291Q	>3.2 ± 0.2	0.051 ± 0.033
S292N	1.6 ± 0.2	0.033 ± 0.016

OGG1, 8-oxoguanine DNA glycosylase.

a Lower estimate is given because of fast substrate cleavage.

b N/c, not cleaved: low cleavage precluded reliable determination of the kinetic parameters.

c Determined at 5-μM enzyme.

**Figure 5:**
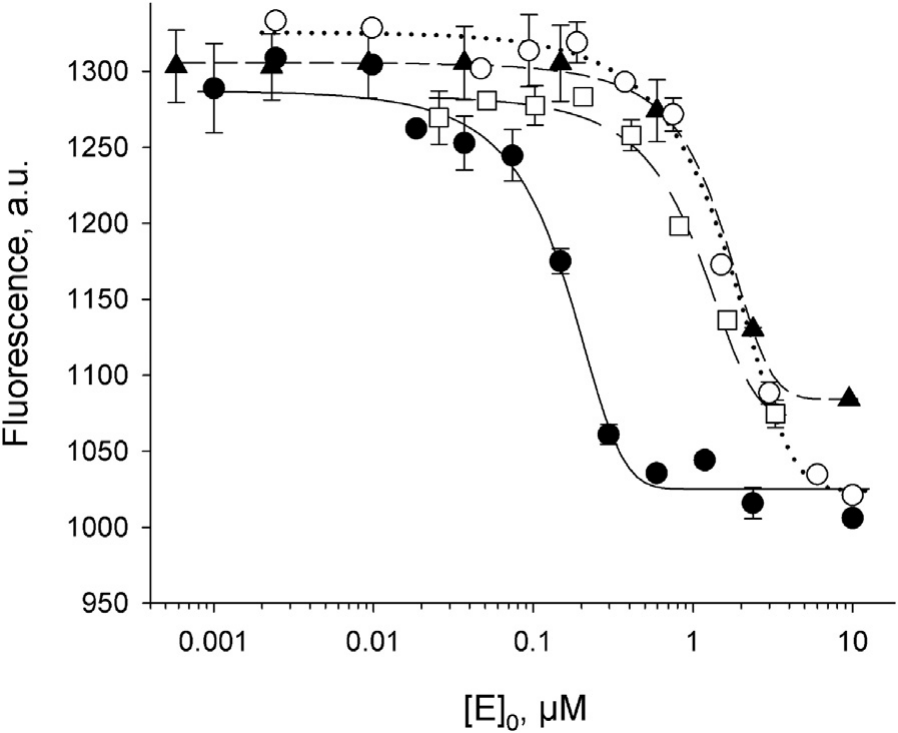
**Binding of OGG1 and two low-activity mutants to THF-containing DNA measured by microscale thermophoresis.***Black circles*, WT OGG1; *white circles*, R161W; *squares*, I145M; *triangles*, S292N. *Symbols* indicate mean ± SE (*n* = 3–4), and *curves* show the fit to the two-state binding model. OGG1, 8-oxoguanine DNA glycosylase; THF, (3-hydroxytetrahydrofuran-2-yl)methyl phosphate.

Loss of the ability to bind DNA can also be a reason for inactivation of OGG1. We have used the microscale thermophoresis technology (51, 52) to compare the affinity of WT OGG1 and four affected or partially affected mutants for a DNA duplex containing (3-hydroxytetrahydrofuran-2-yl)methyl phosphate (THF) and uncleavable AP site analog. As shown in Figure 5 and Fig. S6, WT OGG efficiently formed a complex with THF–DNA (*K*_d_ = 0.14 ± 0.03 μM), with the affinity within the range of values reported in the literature (tens to hundreds nM) that were determined by the gel shift assay and fluorescence titration (49, 53, 54, 55). For I145M, R161W, and S292N, we could not reach binding saturation, but their estimated *K*_d_ values were 7- to 10-fold higher (0.90 ± 0.24 μM, 1.06 ± 0.51 μM, and 1.39 ± 0.45 μM, respectively) than that of the WT. The G202C mutant produced a low signal that could not be reliably quantified, possibly because of fluorescence quenching. It appears that loss of DNA binding at least partly contributes to the decreased activity of OGG1 variants.

### Comparison of the experimental data with predictions

MD does not immediately produce binary results in terms of the mutation effect prediction. We have categorized the mutants according to their position in the distance trees relative to the three-branch point (Fig. 2, *G*–*I*) in two ways: either considering all variants on the distal (*i.e.*, containing the models most remote from the WT) branch as “affected” or all variants on the WT-containing (proximal) branch as “unaffected.” Cohen’s κ was used to compare the classification results with the experimental data (Table 5). The best agreement was observed when the tree was built based on the key geometry parameters, and the longest branch was classified as affected (κ = 0.512 or 0.811 depending on whether the mild R161W and S292N mutants were classified as experimentally affected or not). This came for a price of falsely predicting P291Q as a deleterious mutation. Predictions based on the affected distant branch generally agreed with the experimental data better than predictions based on the unaffected proximal branch because of a lower number of false positives.

**Table 5:** Cohen’s κ for agreement between the predicted and experimentally observed effect of mutations in OGG1

Prediction method	R161W and S292N neutral	R161W and S292 affected
(1) Active site PCA (all), distal branch affected	0.317	0.378
(2) Active site PCA (all), proximal branch unaffected	0.143	0.429
(3) Active site PCA (different from WT), distal branch affected	0.429	0.429
(4) Active site PCA (different from WT), proximal branch unaffected	0.197	0.364
(5) Geometric PCA, distal branch affected	0.811	0.512
(6) Geometric PCA, proximal branch unaffected	0.426	0.429
SIFT	0.340	0.314
FATHMM	0.152	0.317
MutationTaster	0.263	0.208
PROVEAN	0.533	0.553
Combined sequence–based methods	0.519	0.530
Combined sequence–based methods and (5)	0.533	0.553

OGG1, 8-oxoguanine DNA glycosylase; PCA, principal component analysis.

It was also interesting to compare the experimental data with the sequence-based predictions. All four tested algorithms tended to overpredict the number of affected mutants; PROVEAN showed less overprediction and demonstrated better agreement with the experiment (Table 5). Using a combined score from all four algorithms did not improve the overall prediction accuracy. MD-based prediction was superior if R161W and S292N were considered active; if they were counted as affected, PROVEAN and combined sequence–based methods fared better. However, adding the best MD-based method to the combined sequence–based methods improved the prediction accuracy regardless of the R161W/S292N status (Table 5).

## Discussion

The rapid progress of algorithms and hardware for MD has opened the possibility for application of its methods for prediction of effects of mutations on protein function. Although still computationally more expensive than sequence-based methods, MD gives an option of assessing the structural consequences of mutations in detail, which, in turn, allows better segregation of amino acid changes into neutral and deleterious. However, as with any other prediction method, MD results should be benchmarked against real experimental data to ensure their reliability.

We have applied MD to analyze the structures of somatic tumor variants of OGG1, an important DNA repair protein, and to predict their consequences. OGG1 is well characterized structurally, with an extensive set of X-ray structures illuminating its catalytic cycle from the discrimination between normal and damaged DNA to the postexcision steps (43, 44, 56, 57, 58, 59, 60, 61, 62, 63, 64). Based on these structures, several MD and quantum mechanics/molecular mechanics exercises were attempted to investigate the process of dynamic damaged base recognition (45, 46, 65, 66, 67) and catalytic steps (58, 68, 69, 70, 71, 72). Experimental consequences of many site-directed mutations were characterized as part of mechanistic studies on OGG1 activity (43, 44, 45, 46, 62, 73, 74). Good understanding of OGG1 mechanism at both experimental and computational levels makes this enzyme a suitable candidate for attempts to predict the effects of mutations of unknown significance.

In our effort, we have reasoned that the main criterion of the “effect” categorization should be dissimilarity between MD trajectories of the WT protein and a mutant. However, there are many possible ways both to quantify this difference and to limit it to functionally important regions. Knowledge of the mechanism of the protein under study is critical to define such regions. Although we have used PCA and three versions of the “important region,” other metrics and definitions can certainly apply and may aid in better clustering of MD trajectories. However, two observations that we made may be useful to consider when searching for optimal mutation categorization. First, an increase in the number of analyzed variables (the whole active site) produced less-pronounced trajectory resolution and worse correspondence with the experimental data. Second, categorization based on the most distant populations was better than that based on clustering with the WT protein, mostly due to lower overprediction. Overprediction of deleterious effects was also the major factor decreasing the accuracy of sequence-based methods. In our study, the best consistency with the experiment was achieved by MD taking into account three key active site parameters, although this might change with more mutants tested experimentally. As MD improved the output of combined sequence-based methods, it seems likely that MD can indeed be considered a useful addition to the repertoire of tools used to predict functional consequences of amino acid changes in proteins. Our simulation time, 40 ns, is comparatively short by today’s supercomputer standards yet attainable in reasonable time for medium-size molecular systems even with relatively low-end hardware such as graphical processors and thus represents a trade-off between the modeling quality and accessibility.

**Figure 6:**
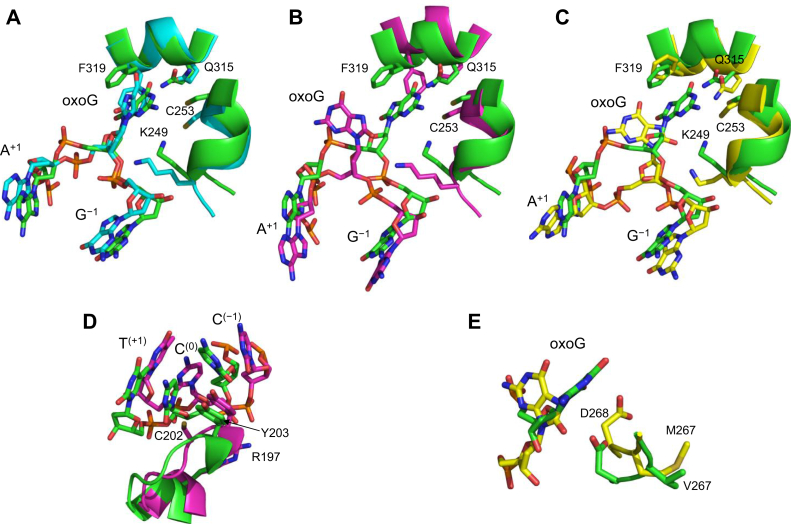
**Effect of mutations on the active site and local conformation of OGG1.***A*–*C*, overlay of the active site of WT OGG1 (*green*) with I145M (*A*, *cyan*), G202C (*B*, *magenta*), and V267M (*C*, *yellow*). *D*, overlay of the region including cytosine opposite the lesion, two flanking bases, and the Tyr203 wedge in WT OGG1 (*green*) and G202C (*magenta*). *E*, overlay of the region including oxoG and the catalytic Asp268 in WT OGG1 (*green*) and V267M (*yellow*). The structures represent the MD snapshots closest to the center of mass of the clusters in the PC1 *versus* PC2 plane (Fig. S2) and thus may be regarded as “typical” for each variant. OGG1, 8-oxoguanine DNA glycosylase; oxoG, 8-oxoguanine; MD, molecular dynamics.

We ran our MD pipeline with 20 OGG1 mutants, of which 19 were never characterized before. To enable the prediction accuracy estimate, we analyzed the activity of 13 of these mutants (plus the WT protein) and obtained more detailed kinetics and binding data for the variants that deviated significantly from the WT. No significant correlation was found between MD predictions and success or failure to produce the mutant protein. Three somatic variants demonstrated significantly impaired activity: I145M, G202C, and, to a lesser degree, V267M. Notably, all these mutants produce very distinct MD trajectories, in which the region conformationally optimal for catalysis was barely populated (Fig. S2). In the OGG1 structure (Fig. 1*B*), Ile145 and Gly202 all lie in a protein lobe that extends into the minor groove, widening it and assisting the insertion of the intercalating residues (Asn149 and Tyr203) that trigger oxoG eversion from the double helix into the enzyme’s active site. Gly202 is located in the αH/αI turn and presses against the sugar ring of the orphaned cytosine, while Ile145 forms part of the hydrophobic core of the lobe. Although these groups do not fall within 5 Å of the oxoG residue, their mutations affect the dynamics of the active site; the most pronounced changes (>36° in the mean angle, *p* < 0.001) were observed for the catalytic Lys249 and oxodG with its adjacent nucleotides (Fig. 6, *A*–*B*). The damaged base lost all stabilizing interactions with Cys253, Gln315, and Phe319 of the damage recognition pocket and was shifted from its position in the WT protein. Locally, the G202C mutation destabilized the cytosine opposite the lesion, as well as two flanking bases, pressing them toward the minor groove (Fig. 6*D*). Val267 is located next to the catalytic Asp268, which stabilizes the positive charge developing on the deoxyribose during base excision, and the V267M mutation causes the Asp268 carboxyl to turn away from the oxodG sugar ring and also disrupting the interactions of oxoG with the base recognition pocket (Fig. 6, *C* and *E*).

Inactivating mutations also significantly impaired DNA binding. Compared with active site disorder, reliable MD prediction of the affinity of protein variants for DNA is more resource-demanding (75). However, as active site disorder contributes to destabilization of the enzyme–substrate precatalytic complex, active site–based predictions are not necessarily limited to the effects on catalytic steps but can be partly related to DNA binding efficiency. Careful selection of the regions of importance outside of the immediate active site may improve the overall prediction reliability.

From the biological standpoint, the “hyperactive” OGG1 mutants we observed are perhaps even more interesting than the inactivating mutants because of their somatic cancer origin. Although the magnitude of the effect is not great, such mutations in DNA repair genes might arise and get fixed because of treatment-driven selection in tumors. The ability of such variants to protect human cells from genotoxicity of cancer drugs merits further attention.

## Experimental procedures

### Model preparation

The atomic structure of human OGG1 (protein data bank [PDB] ID: 1EBM) (43) containing a 15-mer DNA duplex was taken as a reference model. The K249Q inactivation mutation present in 1EBM was manually reverted to Lys249 by editing the PDB file. The backbone of missing residues 80 to 82 was taken from the free OGG1 structure (PDB ID: 1KO9), aligned for the best fit using the Kabsch algorithm, and incorporated into the PDB file. Subsequent modeling was performed in explicit water, so all water molecules found in the crystal unit cells were removed. The final model preparation and refinement was performed in LEaP, a part of the AMBER Tools Package, and the model was minimized in 2500 steps of the steepest descent method followed by 2500 steps of the conjugate gradient method using the SANDER module of the AMBER MD suite (76).

### Automatic mutation selection

The list of sample missense OGG1 mutations was prepared using the custom Python script M3R-PDB (Missense mutation mapper and randomizer for PDB files), available as an open source code from https://doi.org/10.5281/zenodo.3828057. The script connected to the COSMIC database (39) and fetched the full list of missense mutations in the *OGG1* gene. Then, it mapped the mutations onto the known FASTA sequences fetched from the NCBI Reference Sequence Database (77) and determined the original protein sequence, which was then aligned with the OGG1 sequence in 1EBM. Finally, the script randomly picked 20 mutations and output 20 PDB files based on the reference model, each containing a single mutation.

### MD

The PDB model output by M3R-PDB script, as well as the reference model, were prepared, solvated, and verified in LEaP. The explicit TIP3P solvent model was used, and Na^+^ counter ions were added to ensure neutrality. The models were optimized using the SANDER module of the AMBER MD suite in two steps. At the first step, the protein–DNA complex was restrained with the harmonic constant 500 kcal/mol, and only the energy of solvent molecules was minimized in 1000 steps of the steepest descent followed by 1000 steps of conjugate gradients. At the second step, the restrains were removed and the whole system was minimized in 2500 steps of the steepest descent followed by 2500 steps of conjugate gradients. Then the system was gradually heated to 310 K during 200 ps with a 2-fs time step using the ff99SB force field (78) with parmbsc0 corrections (79) using the pmemd module of the AMBER MD suite. The SHAKE algorithm (80) was applied to constrain the bonds involving hydrogens. Heating was performed in Langevin thermostat with a collision frequency of 2 ps^−1^; solvation effects were modeled explicitly using particle mesh Ewald electrostatics algorithm (81). The production run was performed for 40 ns under the same conditions, with snapshots captured every 8 ps, thus producing trajectories of 5000 snapshots each. The trajectories in the mdcrd format were processed by the custom script Mdcrd2pdbs (available as an open source code from https://doi.org/10.5281/zenodo.3828060), stripping most of the solvent except the water molecules located within 5.0 Å of the oxoG residue for at least 10% of the trajectory.

### Trajectory analysis

The resulting all-atom trajectories were processed with the MDTRA software package (82) to extract the required geometric parameters, which were further analyzed using R scripts. The last 2500 snapshots were included in the analysis. The PCA was performed for the combined set of all trajectories. The PC1 *versus* PC2 plane was partitioned into a 10 × 10 equidistant grid, and Bhattacharyya coefficients $BC=\overset{n}{\underset{i=1}{\sum }}\sqrt{p_{i}q_{i}}$ (*n*, total number of bins; *p*_*i*_ and *q*_*i*_, numbers of occurrences of two samples in the *i*^th^ bin) were calculated over this partition for every pair of trajectories to provide a measure of the overlap between them (83). Because by definition 0 ≤ *BC* ≤ *N* (*N* = 2500, the total number of points in any trajectory), *N* − *BC* was taken as the distance measure. The pairwise distance matrix was converted to a tree using the unweighted PGMA approach and visualized using Interactive Tree Of Life (84).

### Oligonucleotides and enzymes

Oligonucleotides 5′-CTCTCCCTTCXCTCCTTTCCTCT-3′ (X = oxoG or THF) and the complementary strand 5′-AGAGGAAAGGAGCGAAGGGAGAG-3′ were synthesized in-house from commercially available phosphoramidites (Glen Research, Sterling, VA). The oxoG-containing strand was 5′-labeled using γ[^32^P]ATP (SB RAS ICBFM Laboratory of Biotechnology) and phage T4 polynucleotide kinase (SibEnzyme, Novosibirsk, Russia) according to the manufacturer’s protocol, purified on NENSORB C_18_ sorbent (DuPont, Wilmington, DE) and annealed to an 1.5-fold molar excess of the complementary strand. The THF-containing oligonucleotide carried a fluorescein residue at the 5′-end. Full-length OGG1 mutants carrying an N-terminal His_6_-tag were produced using the Q5 Site-Directed Mutagenesis Kit (New England Biolabs, Beverly, MA) with the pET-15b-OGG1 plasmid (53) as a template. The target mutations were confirmed by Sanger sequencing. The plasmids were transformed into Rosetta 2(DE3) *E. coli* (Merck Millipore, Burlington, MA). WT and mutant OGG1 were overexpressed and purified essentially as described (53). Seven of 20 selected mutants were refractory to the purification attempts, yielding no visible induction or no band of the expected mobility in the soluble fraction in a screen that involved four DE3 bacterial strains (BL21, Rosetta 2, SoluBL21, and ArcticExpress), two induction temperatures (15 °C and 37 °C), two IPTG concentrations (50 and 1000 μM), and two induction times (3 h and overnight). The total protein concentration in the purified preparations was determined using the calculated extinction coefficient at 280 nm (85). The concentration of the active WT enzyme and, when possible, the mutants was estimated from burst-phase experiments as outlined below; the fraction of the active enzyme varied from ∼35% to ∼90%. Recombinant human APEX1 was purified as described (48).

### Enzyme kinetics

For single time-point experiments and turnover rate constant (*k*_3_) determination, the reaction mixture contained 100-nM ^32^P-labeled substrate duplex, 50-mM Tris HCl (pH 7.5), 100-mM NaCl, 1-mM EDTA, 1-mM DTT, and 10- to 15-nM OGG1 (WT or mutant). The reaction was allowed to proceed at 37 °C for 30 min (single time-point assay), 0 to 60 min (*k*_3_ experiments for H111R, G202C, R206C, S292N), or 0 to 20 min (*k*_3_ experiments for all other variants). To determine the glycosidic bond cleavage rate constant (*k*_2_), the substrate concentration was 20 nM, and the OGG1 concentration, 800 nM (additionally, 5000 nM for G202C), and the reaction was carried out at 15 °C for 0 to 120 min (G202C), 0 to 60 min (R161W, V267M, and S292N), or 0 to 30 min (all other variants). In all cases, 10-μl aliquots were withdrawn at indicated times (30 min for the single time point assay) and quenched by mixing with 1 μl of 1-M NaOH and heating for 3 min at 95 °C. The samples were neutralized with an equimolar amount of HCl and mixed with gel-loading dye (20-mM EDTA, 0.1% xylene cyanol, 0.1% bromophenol blue in 80% formamide). The reaction products were resolved by gel electrophoresis in 20% polyacrylamide/7.2 M urea and quantified by phosphorimaging (Typhoon FLA 9500, GE Healthcare, Chicago, IL). The apparent rate constants for the rate-limiting product release scheme (48, 50) were calculated from 3 to 5 independent experiments using SigmaPlot v11.0 (Systat Software, San Jose, CA). Each replicate was performed with independently prepared protein dilutions. The values for apparent *k*_2_ constants were determined by fitting the cleavage time dependencies to the equation $\left[\text{P}\right]={\left[\text{P}\right]}_{\text{max}}\left(1-e^{-k_{2\;\text{app}}t}\right)$, where [P]_max_ is the maximal product concentration and *t* is time. The values for apparent *k*_3_ constants were determined by fitting the cleavage time dependencies to the equation $\left[\text{P}\right]=A_{0}\left(1-e^{-kt}\right)+k_{3\text{ app}}{\left[\text{E}\right]}_{0}t$, where *A*_0_ is the burst phase amplitude, *k* is the observed exponential phase constant, [E]_0_ is the enzyme concentration, and *t* is time.

### Protein thermal denaturation

OGG1 melting profiles were analyzed by native differential scanning fluorimetry, following the ratio of tryptophan fluorescence at λ_em_ = 350 nm and λ_em_ = 330 nm (λ_ex_ = 280 nm) using the Tycho NT.6 capillary fluorimeter (NanoTemper Technologies, Munich, Germany). The change in the intrinsic fluorescence was monitored in the temperature range 35 to 95 °C at a rate of 30 °C/min. All protein solutions contained OGG1 (WT or mutant), 20-mM Na phosphate buffer (pH 7.5), 400-mM NaCl, 1-mM DTT, 1-mM EDTA, and 50% glycerol (v/v).

### Protein–DNA binding

Binding of OGG1 and its mutants to a fluorescent THF-containing duplex was measured by microscale thermophoresis (51). All reaction mixtures with a final volume of 10 μl consisted of labeled DNA duplex (100 nM), 0.0185- to 3.28-μM unlabeled protein, and 10-mM Na phosphate (pH 7.5). Glycerol concentration in all reaction mixtures was adjusted to 7%. Measurements were carried out using standard capillaries in the Monolith NT.115 device (NanoTemper Technologies) equipped with a red/green detection channel and medium infrared laser power. The binding constants were calculated from two independent experiments using SigmaPlot v11.0.

## Data availability

The software is available as a source code from https://doi.org/10.5281/zenodo.3828057 (M3R-PDB) and https://doi.org/10.5281/zenodo.3828060 (mdcrd2pdbs). MD trajectories are available from Dmitry O. Zharkov upon request (dzharkov@niboch.nsc.ru). All other data are contained within the manuscript.

## Conflict of interest

The authors declare that they have no conflicts of interest with the contents of this article.

## References

[1] Friedberg E.C., Walker G.C., Siede W., Wood R.D., Schultz R.A., Ellenberger T. (2006). DNA Repair and Mutagenesis.

[2] Zharkov D.O. (2008). Base excision DNA repair. Cell Mol. Life Sci..

[3] Lawley P.D., Phillips D.H. (1996). DNA adducts from chemotherapeutic agents. Mutat. Res..

[4] Fong P.C., Boss D.S., Yap T.A., Tutt A., Wu P., Mergui-Roelvink M., Mortimer P., Swaisland H., Lau A., O'Connor M.J., Ashworth A., Carmichael J., Kaye S.B., Schellens J.H.M., de Bono J.S. (2009). Inhibition of poly(ADP-ribose) polymerase in tumors from *BRCA* mutation carriers. N. Engl. J. Med..

[5] Curtin N.J., Szabo C. (2013). Therapeutic applications of PARP inhibitors: anticancer therapy and beyond. Mol. Aspects Med..

[6] Kaina B., Margison G.P., Christmann M. (2010). Targeting *O*^6^-methylguanine-DNA methyltransferase with specific inhibitors as a strategy in cancer therapy. Cell Mol. Life Sci..

[7] Blumenthal D.T., Rankin C., Stelzer K.J., Spence A.M., Sloan A.E., Moore D.F., Padula G.D.A., Schulman S.B., Wade M.L., Rushing E.J. (2015). A phase III study of radiation therapy (RT) and O^6^-benzylguanine + BCNU versus RT and BCNU alone and methylation status in newly diagnosed glioblastoma and gliosarcoma: Southwest Oncology Group (SWOG) study S0001. Int. J. Clin. Oncol..

[8] Burrell R.A., McGranahan N., Bartek J., Swanton C. (2013). The causes and consequences of genetic heterogeneity in cancer evolution. Nature.

[9] Beckman R.A., Loeb L.A. (2017). Evolutionary dynamics and significance of multiple subclonal mutations in cancer. DNA Repair.

[10] Chae Y.K., Anker J.F., Carneiro B.A., Chandra S., Kaplan J., Kalyan A., Santa-Maria C.A., Platanias L.C., Giles F.J. (2016). Genomic landscape of DNA repair genes in cancer. Oncotarget.

[11] Germano G., Lamba S., Rospo G., Barault L., Magrì A., Maione F., Russo M., Crisafulli G., Bartolini A., Lerda G., Siravegna G., Mussolin B., Frapolli R., Montone M., Morano F. (2017). Inactivation of DNA repair triggers neoantigen generation and impairs tumour growth. Nature.

[12] Kamps R., Brandão R.D., van den Bosch B.J., Paulussen A.D.C., Xanthoulea S., Blok M.J., Romano A. (2017). Next-generation sequencing in oncology: genetic diagnosis, risk prediction and cancer classification. Int. J. Mol. Sci..

[13] Castellana S., Mazza T. (2013). Congruency in the prediction of pathogenic missense mutations: state-of-the-art web-based tools. Brief Bioinform..

[14] Khafizov K., Ivanov M.V., Glazova O.V., Kovalenko S.P. (2015). Computational approaches to study the effects of small genomic variations. J. Mol. Model..

[15] Martelotto L.G., Ng C.K.Y., De Filippo M.R., Zhang Y., Piscuoglio S., Lim R.S., Shen R., Norton L., Reis-Filho J.S., Weigelt B. (2014). Benchmarking mutation effect prediction algorithms using functionally validated cancer-related missense mutations. Genome Biol..

[16] Ng P.C., Henikoff S. (2006). Predicting the effects of amino acid substitutions on protein function. Annu. Rev. Genomics Hum. Genet..

[17] Carter H., Chen S., Isik L., Tyekucheva S., Velculescu V.E., Kinzler K.W., Vogelstein B., Karchin R. (2009). Cancer-specific high-throughput annotation of somatic mutations: computational prediction of driver missense mutations. Cancer Res..

[18] Kumar P., Henikoff S., Ng P.C. (2009). Predicting the effects of coding non-synonymous variants on protein function using the SIFT algorithm. Nat. Protoc..

[19] Adzhubei I.A., Schmidt S., Peshkin L., Ramensky V.E., Gerasimova A., Bork P., Kondrashov A.S., Sunyaev S.R. (2010). A method and server for predicting damaging missense mutations. Nat. Methods.

[20] González-Pérez A., López-Bigas N. (2011). Improving the assessment of the outcome of nonsynonymous SNVs with a consensus deleteriousness score, condel. Am. J. Hum. Genet..

[21] Reva B., Antipin Y., Sander C. (2011). Predicting the functional impact of protein mutations: application to cancer genomics. Nucleic Acids Res..

[22] Choi Y., Sims G.E., Murphy S., Miller J.R., Chan A.P. (2012). Predicting the functional effect of amino acid substitutions and indels. PLoS One.

[23] Sim N.-L., Kumar P., Hu J., Henikoff S., Schneider G., Ng P.C. (2012). SIFT web server: predicting effects of amino acid substitutions on proteins. Nucleic Acids Res..

[24] Shihab H.A., Gough J., Cooper D.N., Stenson P.D., Barker G.L.A., Edwards K.J., Day I.N.M., Gaunt T.R. (2013). Predicting the functional, molecular, and phenotypic consequences of amino acid substitutions using hidden Markov models. Hum. Mutat..

[25] Schwarz J.M., Cooper D.N., Schuelke M., Seelow D. (2014). MutationTaster2: mutation prediction for the deep-sequencing age. Nat. Methods.

[26] Wong K.-C., Zhang Z. (2014). SNPdryad: predicting deleterious non-synonymous human SNPs using only orthologous protein sequences. Bioinformatics.

[27] Raimondi D., Gazzo A.M., Rooman M., Lenaerts T., Vranken W.F. (2016). Multilevel biological characterization of exomic variants at the protein level significantly improves the identification of their deleterious effects. Bioinformatics.

[28] Pejaver V., Mooney S.D., Radivojac P. (2017). Missense variant pathogenicity predictors generalize well across a range of function-specific prediction challenges. Hum. Mutat..

[29] Dehghanpoor R., Ricks E., Hursh K., Gunderson S., Farhoodi R., Haspel N., Hutchinson B., Jagodzinski F. (2018). Predicting the effect of single and multiple mutations on protein structural stability. Molecules.

[30] Kucukkal T.G., Petukh M., Li L., Alexov E. (2015). Structural and physico-chemical effects of disease and non-disease nsSNPs on proteins. Curr. Opin. Struct. Biol..

[31] Sneha P., Doss C.G.P. (2016). Molecular dynamics: new frontier in personalized medicine. Adv. Protein Chem. Struct. Biol..

[32] Gibbons D.L., Pricl S., Posocco P., Laurini E., Fermeglia M., Sun H., Talpaz M., Donato N., Quintás-Cardama A. (2014). Molecular dynamics reveal BCR-ABL1 polymutants as a unique mechanism of resistance to PAN-BCR-ABL1 kinase inhibitor therapy. Proc. Natl. Acad. Sci. U. S. A..

[33] Oliver C., Timson D.J. (2017). *In silico* prediction of the effects of mutations in the human triose phosphate isomerase gene: towards a predictive framework for TPI deficiency. Eur. J. Med. Genet..

[34] Wu M., Zhang Z., Che W. (2008). Suppression of a DNA base excision repair gene, hOGG1, increases bleomycin sensitivity of human lung cancer cell line. Toxicol. Appl. Pharmacol..

[35] Kobune M., Xu Y., Baum C., Kelley M.R., Williams D.A. (2001). Retrovirus-mediated expression of the base excision repair proteins, formamidopyrimidine DNA glycosylase or human oxoguanine DNA glycosylase, protects hematopoietic cells from *N,N′,N*″-triethylenethiophosphoramide (thioTEPA)-induced toxicity *in vitro* and *in vivo*. Cancer Res..

[36] Xu Y., Hansen W.K., Rosenquist T.A., Williams D.A., Limp-Foster M., Kelley M.R. (2001). Protection of mammalian cells against chemotherapeutic agents thiotepa, 1,3-*N,N*′-bis(2-chloroethyl)-*N*-nitrosourea, and mafosfamide using the DNA base excision repair genes Fpg and α-hOgg1: implications for protective gene therapy applications. J. Pharmacol. Exp. Ther..

[37] He Y.-H., Xu Y., Kobune M., Wu M., Kelley M.R., Martin W.J. (2002). *Escherichia coli* FPG and human OGG1 reduce DNA damage and cytotoxicity by BCNU in human lung cells. Am. J. Physiol. Lung Cell Mol. Physiol..

[38] Preston T.J., Henderson J.T., McCallum G.P., Wells P.G. (2009). Base excision repair of reactive oxygen species-initiated 7,8-dihydro-8-oxo-2′-deoxyguanosine inhibits the cytotoxicity of platinum anticancer drugs. Mol. Cancer Ther..

[39] Tate J.G., Bamford S., Jubb H.C., Sondka Z., Beare D.M., Bindal N., Boutselakis H., Cole C.G., Creatore C., Dawson E., Fish P., Harsha B., Hathaway C., Jupe S.C., Kok C.Y. (2019). COSMIC: the catalogue of somatic mutations in cancer. Nucleic Acids Res..

[40] McHugh M.L. (2012). Interrater reliability: the kappa statistic. Biochem. Med..

[41] Kohno T., Shinmura K., Tosaka M., Tani M., Kim S.-R., Sugimura H., Nohmi T., Kasai H., Yokota J. (1998). Genetic polymorphisms and alternative splicing of the *hOGG1* gene, that is involved in the repair of 8-hydroxyguanine in damaged DNA. Oncogene.

[42] Boldinova E.O., Khairullin R.F., Makarova A.V., Zharkov D.O. (2019). Isoforms of base excision repair enzymes produced by alternative splicing. Int. J. Mol. Sci..

[43] Bruner S.D., Norman D.P.G., Verdine G.L. (2000). Structural basis for recognition and repair of the endogenous mutagen 8-oxoguanine in DNA. Nature.

[44] Norman D.P.G., Chung S.J., Verdine G.L. (2003). Structural and biochemical exploration of a critical amino acid in human 8-oxoguanine glycosylase. Biochemistry.

[45] Lukina M.V., Popov A.V., Koval V.V., Vorobjev Y.N., Fedorova O.S., Zharkov D.O. (2013). DNA damage processing by human 8-oxoguanine-DNA glycosylase mutants with the occluded active site. J. Biol. Chem..

[46] Popov A.V., Yudkina A.V., Vorobjev Y.N., Zharkov D.O. (2020). Catalytically competent conformation of the active site of human 8-oxoguanine–DNA glycosylase. Biochemistry (Mosc).

[47] Bjørås M., Luna L., Johnsen B., Hoff E., Haug T., Rognes T., Seeberg E. (1997). Opposite base-dependent reactions of a human base excision repair enzyme on DNA containing 7,8-dihydro-8-oxoguanine and abasic sites. EMBO J..

[48] Sidorenko V.S., Nevinsky G.A., Zharkov D.O. (2007). Mechanism of interaction between human 8-oxoguanine-DNA glycosylase and AP endonuclease. DNA Repair.

[49] Esadze A., Rodriguez G., Cravens S.L., Stivers J.T. (2017). AP-endonuclease 1 accelerates turnover of human 8-oxoguanine DNA glycosylase by preventing retrograde binding to the abasic-site product. Biochemistry.

[50] Porello S.L., Leyes A.E., David S.S. (1998). Single-turnover and pre-steady-state kinetics of the reaction of the adenine glycosylase MutY with mismatch-containing DNA substrates. Biochemistry.

[51] Wienken C.J., Baaske P., Rothbauer U., Braun D., Duhr S. (2010). Protein-binding assays in biological liquids using microscale thermophoresis. Nat. Commun..

[52] Seidel S.A.I., Dijkman P.M., Lea W.A., van den Bogaart G., Jerabek-Willemsen M., Lazic A., Joseph J.S., Srinivasan P., Baaske P., Simeonov A., Katritch I., Melo F.A., Ladbury J.E., Schreiber G., Watts A. (2013). Microscale thermophoresis quantifies biomolecular interactions under previously challenging conditions. Methods.

[53] Kuznetsov N.A., Koval V.V., Zharkov D.O., Nevinsky G.A., Douglas K.T., Fedorova O.S. (2005). Kinetics of substrate recognition and cleavage by human 8-oxoguanine-DNA glycosylase. Nucleic Acids Res..

[54] Hill J.W., Evans M.K. (2006). Dimerization and opposite base-dependent catalytic impairment of polymorphic S326C OGG1 glycosylase. Nucleic Acids Res..

[55] Sidorenko V.S., Mechetin G.V., Nevinsky G.A., Zharkov D.O. (2008). Ionic strength and magnesium affect the specificity of *Escherichia coli* and human 8-oxoguanine-DNA glycosylases. FEBS J..

[56] Norman D.P.G., Bruner S.D., Verdine G.L. (2001). Coupling of substrate recognition and catalysis by a human base-excision DNA repair protein. J. Am. Chem. Soc..

[57] Bjørås M., Seeberg E., Luna L., Pearl L.H., Barrett T.E. (2002). Reciprocal “flipping” underlies substrate recognition and catalytic activation by the human 8-oxo-guanine DNA glycosylase. J. Mol. Biol..

[58] Fromme J.C., Bruner S.D., Yang W., Karplus M., Verdine G.L. (2003). Product-assisted catalysis in base-excision DNA repair. Nat. Struct. Biol..

[59] Chung S.J., Verdine G.L. (2004). Structures of end products resulting from lesion processing by a DNA glycosylase/lyase. Chem. Biol..

[60] Banerjee A., Yang W., Karplus M., Verdine G.L. (2005). Structure of a repair enzyme interrogating undamaged DNA elucidates recognition of damaged DNA. Nature.

[61] Banerjee A., Verdine G.L. (2006). A nucleobase lesion remodels the interaction of its normal neighbor in a DNA glycosylase complex. Proc. Natl. Acad. Sci. U. S. A..

[62] Radom C.T., Banerjee A., Verdine G.L. (2007). Structural characterization of human 8-oxoguanine DNA glycosylase variants bearing active site mutations. J. Biol. Chem..

[63] Dalhus B., Forsbring M., Helle I.H., Vik E.S., Forstrøm R.J., Backe P.H., Alseth I., Bjørås M. (2011). Separation-of-function mutants unravel the dual-reaction mode of human 8-oxoguanine DNA glycosylase. Structure.

[64] Crenshaw C.M., Nam K., Oo K., Kutchukian P.S., Bowman B.R., Karplus M., Verdine G.L. (2012). Enforced presentation of an extrahelical guanine to the lesion recognition pocket of human 8-oxoguanine glycosylase, hOGG1. J. Biol. Chem..

[65] Lee S., Radom C.T., Verdine G.L. (2008). Trapping and structural elucidation of a very advanced intermediate in the lesion-extrusion pathway of hOGG1. J. Am. Chem. Soc..

[66] Li H., Endutkin A.V., Bergonzo C., Fu L., Grollman A.P., Zharkov D.O., Simmerling C. (2017). DNA deformation-coupled recognition of 8-oxoguanine: conformational kinetic gating in human DNA glycosylase. J. Am. Chem. Soc..

[67] Sowlati-Hashjin S., Wetmore S.D. (2018). Structural insight into the discrimination between 8-oxoguanine glycosidic conformers by DNA repair enzymes: a molecular dynamics study of human oxoguanine glycosylase 1 and formamidopyrimidine-DNA glycosylase. Biochemistry.

[68] Schyman P., Danielsson J., Pinak M., Laaksonen A. (2005). Theoretical study of the human DNA repair protein HOGG1 activity. J. Phys. Chem. A.

[69] Calvaresi M., Bottoni A., Garavelli M. (2007). Computational clues for a new mechanism in the glycosylase activity of the human DNA repair protein hOGG1. A generalized paradigm for purine-repairing systems?. J. Phys. Chem. B.

[70] Kellie J.L., Wetmore S.D. (2012). Mechanistic and conformational flexibility of the covalent linkage formed during β-lyase activity on an AP-site: application to hOgg1. J. Phys. Chem. B.

[71] Kellie J.L., Wilson K.A., Wetmore S.D. (2015). An ONIOM and MD investigation of possible monofunctional activity of human 8-oxoguanine–DNA glycosylase (hOgg1). J. Phys. Chem. B.

[72] Sadeghian K., Ochsenfeld C. (2015). Unraveling the base excision repair mechanism of human DNA glycosylase. J. Am. Chem. Soc..

[73] Lu R., Nash H.M., Verdine G.L. (1997). A mammalian DNA repair enzyme that excises oxidatively damaged guanines maps to a locus frequently lost in lung cancer. Curr. Biol..

[74] Tyugashev T.E., Vorobjev Y.N., Kuznetsova A.A., Lukina M.V., Kuznetsov N.A., Fedorova O.S. (2019). Roles of active-site amino acid residues in specific recognition of DNA lesions by human 8-oxoguanine-DNA glycosylase (OGG1). J. Phys. Chem. B.

[75] Liu L.A., Bradley P. (2012). Atomistic modeling of protein–DNA interaction specificity: progress and applications. Curr. Opin. Struct. Biol..

[76] Case D.A., Darden T.A., Cheatham T.E., Simmerling C.L., Wang J., Duke R.E., Luo R., Walker R.C., Zhang W., Merz K.M., Roberts B., Wang B., Hayik S., Roitberg A., Seabra G. (2010). AMBER 11.

[77] Geer L.Y., Marchler-Bauer A., Geer R.C., Han L., He J., He S., Liu C., Shi W., Bryant S.H. (2010). The NCBI BioSystems database. Nucleic Acids Res..

[78] Hornak V., Abel R., Okur A., Strockbine B., Roitberg A., Simmerling C. (2006). Comparison of multiple Amber force fields and development of improved protein backbone parameters. Proteins.

[79] Pérez A., Marchán I., Svozil D., Sponer J., Cheatham T.E., Laughton C.A., Orozco M. (2007). Refinement of the AMBER force field for nucleic acids: improving the description of α/γ conformers. Biophys. J..

[80] Ryckaert J.-P., Ciccotti G., Berendsen H.J.C. (1977). Numerical integration of the Cartesian equations of motion of a system with constraints: molecular dynamics of *n*-alkanes. J. Comput. Phys..

[81] Darden T., York D., Pedersen L. (1993). Particle mesh Ewald: an *N*•log(*N*) method for Ewald sums in large systems. J. Chem. Phys..

[82] Popov A.V., Vorobjev Y.N., Zharkov D.O. (2013). MDTRA: a molecular dynamics trajectory analyzer with a graphical user interface. J. Comput. Chem..

[83] Deza M.M., Deza E. (2014). Encyclopedia of Distances.

[84] Letunic I., Bork P. (2016). Interactive tree of life (iTOL) v3: an online tool for the display and annotation of phylogenetic and other trees. Nucleic Acids Res..

[85] Gill S.C., von Hippel P.H. (1989). Calculation of protein extinction coefficients from amino acid sequence data. Anal. Biochem..

[86] Chevillard S., Radicella J.P., Levalois C., Lebeau J., Poupon M.-F., Oudard S., Dutrillaux B., Boiteux S. (1998). Mutations in *OGG1*, a gene involved in the repair of oxidative DNA damage, are found in human lung and kidney tumours. Oncogene.

[87] Anderson P.C., Daggett V. (2009). The R46Q, R131Q and R154H polymorphs of human DNA glycosylase/β-lyase hOgg1 severely distort the active site and DNA recognition site but do not cause unfolding. J. Am. Chem. Soc..

[88] Hyun J.-W., Choi J.-Y., Zeng H.-H., Lee Y.-S., Kim H.-S., Yoon S.-H., Chung M.-H. (2000). Leukemic cell line, KG-1 has a functional loss of hOGG1 enzyme due to a point mutation and 8-hydroxydeoxyguanosine can kill KG-1. Oncogene.

[89] Hyun J.-W., Cheon G.-J., Kim H.-S., Lee Y.-S., Choi E.-Y., Yoon B.-H., Kim J.-S., Chung M.-H. (2002). Radiation sensitivity depends on OGG1 activity status in human leukemia cell lines. Free Radic. Biol. Med..

[90] Hill J.W., Evans M.K. (2007). A novel R229Q OGG1 polymorphism results in a thermolabile enzyme that sensitizes KG-1 leukemia cells to DNA damaging agents. Cancer Detect. Prev..

