# Supplementary material for: Molecular dynamics approach to identification of new OGG1 cancer-associated somatic variants with impaired activity
Source: J Biol Chem. 2021 Jan 7;296:100229. doi: 10.1074/jbc.RA120.014455 (PMC7948927; doi:10.1074/jbc.RA120.014455)
Supplement: Supplementary Figures and Tables [file mmc1.pdf]

SUPPORTING INFORMATION FOR

Molecular dynamics approach to identification of new OGG1 cancer-associated somatic variants with impaired activity

Alexander V. Popov[1,†,*], Anton V. Endutkin[1,†], Darya D. Yatsenko[1,2], Anna V. Yudkina[1],
Alexander E. Barmatov[1], Kristina A. Makasheva[2], Darya Yu. Raspopova[2], Evgeniia A. Diatlova[1],
Dmitry O. Zharkov[1,2,*]

[1]Laboratory of Genome and Protein Engineering, SB RAS Institute of Chemical Biology and Fundamental Medicine, Novosibirsk, Russia
[2]Novosibirsk State University, Novosibirsk, Russia

[†]These authors contributed equally to this work

[*]Corresponding authors: Alexander V. Popov and Dmitry O. Zharkov

E-mail: apopov@niboch.nsc.ru, dzharkov@niboch.nsc.ru

Supporting Table S1. OGG1 mutations with their effects predicted from the sequence

Variant	SIFT	FATHMM	MutationTaster	PROVEAN
R4C	affects protein function	tolerated	polymorphism	neutral
R10H	tolerated	tolerated	polymorphism	neutral
P29T	tolerated	tolerated	disease causing	neutral
R46L	affects protein function	tolerated	disease causing	deleterious
Q62H	tolerated	tolerated	disease causing	neutral
A85T	tolerated	tolerated	polymorphism	neutral
R87K	tolerated	tolerated	polymorphism	neutral
P88L	tolerated	tolerated	disease causing	neutral
E92D	tolerated	tolerated	disease causing	neutral
R97C	affects protein function	tolerated	polymorphism	deleterious
R97L	tolerated	tolerated	polymorphism	neutral
H111R	tolerated	tolerated	polymorphism	neutral
S115F	affects protein function	tolerated	polymorphism	deleterious
V116E	tolerated	tolerated	polymorphism	neutral
D117Y	affects protein function	tolerated	disease causing	deleterious
S118F	affects protein function	tolerated	disease causing	deleterious
G129C	affects protein function	tolerated	disease causing	deleterious
R131G	affects protein function	damaging	disease causing	deleterious
I145M	affects protein function	tolerated	disease causing	deleterious
N151S	affects protein function	tolerated	disease causing	deleterious
A153T	tolerated	damaging	polymorphism	neutral
R161W	affects protein function	damaging	disease causing	deleterious
D175N	tolerated	tolerated	polymorphism	neutral
G202C	affects protein function	damaging	disease causing	deleterious
R206C	affects protein function	damaging	disease causing	deleterious
R213Q	tolerated	damaging	polymorphism	neutral
Q226H	tolerated	tolerated	disease causing	neutral
Q263H	affects protein function	tolerated	disease causing	neutral
P266L	affects protein function	damaging	disease causing	deleterious
V267M	affects protein function	tolerated	disease causing	deleterious
R277H	affects protein function	damaging	disease causing	deleterious
T285M	tolerated	tolerated	polymorphism	neutral
G290E	affects protein function	tolerated	polymorphism	neutral
P291Q	affects protein function	tolerated	polymorphism	neutral
S292N	tolerated	tolerated	polymorphism	neutral
R324H	affects protein function	tolerated	polymorphism	deleterious

Supporting Table S1 (continued). UNG mutations with their effects predicted from the sequence

Variant	SIFT	FATHMM	MutationTaster	PROVEAN
G2V	affects protein function	tolerated	disease causing	neutral
P18R	affects protein function	tolerated	polymorphism	neutral
L29P	tolerated	tolerated	polymorphism	neutral
K40N	affects protein function	tolerated	disease causing	neutral
A42V	affects protein function	tolerated	polymorphism	neutral
P43L	affects protein function	tolerated	polymorphism	neutral
P56L	affects protein function	tolerated	disease causing	neutral
D63G	tolerated	tolerated	polymorphism	neutral
N68S	affects protein function	tolerated	disease causing	neutral
K69N	affects protein function	tolerated	disease causing	neutral
A78D	affects protein function	tolerated	disease causing	neutral
K90M	affects protein function	tolerated	disease causing	neutral
L93V	affects protein function	damaging	disease causing	neutral
G95R	tolerated	tolerated	polymorphism	neutral
G98E	tolerated	tolerated	disease causing	neutral
F108C	affects protein function	tolerated	disease causing	deleterious
K114N	tolerated	tolerated	disease causing	neutral
Y119H	affects protein function	tolerated	disease causing	deleterious
T127I	affects protein function	tolerated	disease causing	deleterious
W128L	tolerated	tolerated	disease causing	deleterious
D133Y	affects protein function	tolerated	disease causing	deleterious
H154Y	affects protein function	tolerated	disease causing	deleterious
H154Q	affects protein function	tolerated	disease causing	deleterious
G155R	affects protein function	damaging	disease causing	deleterious
V160F	affects protein function	tolerated	disease causing	deleterious
S178P	affects protein function	tolerated	disease causing	neutral
S178Y	affects protein function	tolerated	disease causing	deleterious
S178F	affects protein function	tolerated	disease causing	deleterious
T179I	affects protein function	tolerated	disease causing	deleterious
N204D	affects protein function	damaging	disease causing	deleterious
A205T	tolerated	tolerated	disease causing	deleterious
V209I	affects protein function	tolerated	disease causing	neutral
A211V	affects protein function	tolerated	disease causing	deleterious
N215K	affects protein function	tolerated	disease causing	deleterious
S216C	affects protein function	damaging	disease causing	deleterious
S216F	affects protein function	damaging	disease causing	deleterious
R260W	affects protein function	tolerated	disease causing	deleterious
R260Q	tolerated	tolerated	disease causing	deleterious
V263G	affects protein function	tolerated	disease causing	deleterious
Q265L	affects protein function	tolerated	disease causing	deleterious

H268R	affects protein function	damaging	disease causing	deleterious
P269S	affects protein function	tolerated	disease causing	deleterious
L272F	affects protein function	tolerated	disease causing	deleterious
G295D	affects protein function	tolerated	disease causing	deleterious

Supporting Figure legends

Supporting Fig. S1. RMSD of the backbone atoms in the simulated trajectories of wild-type OGG1 and OGG1 mutants.

Supporting Fig. S2. Comparison of the populations of wild-type OGG1 (*blue dots*) and each mutant variant (*red dots*) in the PC1 vs PC2 coordinates derived from the critical geometric parameters of the active site (as in Figure 2C, F, and I).

Supporting Fig. S3. Images of Coomassie Blue-stained gels after SDS-PAGE of the preparations of wild-type and mutant OGG1 proteins. Molecular weight markers: PageRuler Prestained Protein Ladder (ThermoFisher Scientific).

Supporting Fig. S4. Time courses of the substrate cleavage by OGG variants from which k_2 values reported in Table 4 were determined. In the G202C panel, *black circles* show the reaction at 800 nM enzyme, and *red circles*, at 5000 nM enzyme.

Supporting Fig. S5. Time courses of the substrate cleavage by OGG variants from which k_3 values reported in Table 4 were determined.

Supporting Fig. S6. A, microscale thermophoresis traces from a representative experiment with wild-type OGG1 binding the THF:C oligonucleotide duplex. **B,** binding of wild-type OGG1 to the THF:C duplex (*black circles*) and undamaged A:T duplex (*white circles*). Symbols indicate mean ± SE ($n = 4$ for THF:C, $n = 2$ for A:T), curves show the fit to the two-state binding model.

Supporting Figure 1 (see next page)

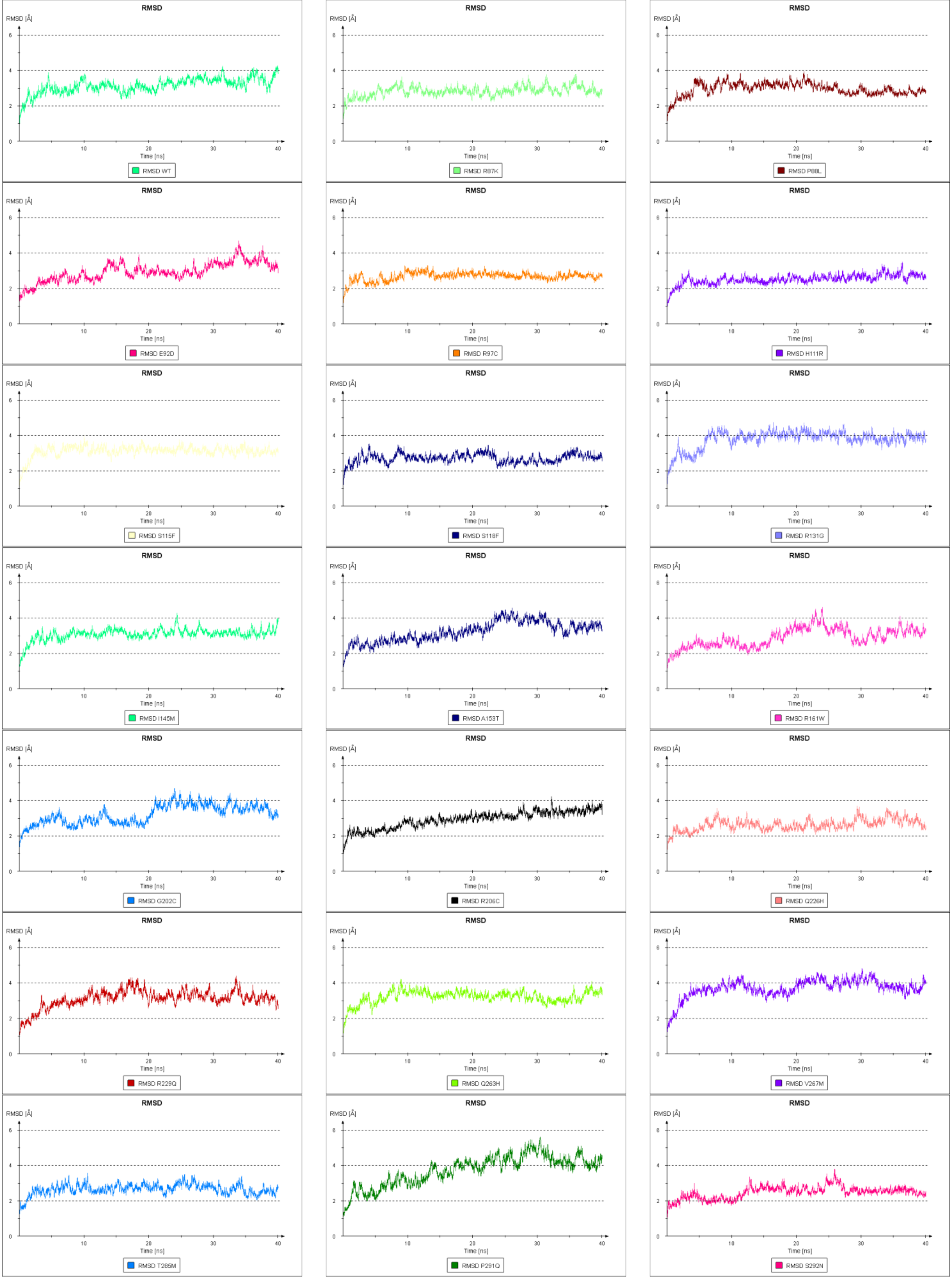

RMSD
RMSD [Å]
0
2
4
6
10
20
30
40
Time [ns]
RMSD WT
RMSD R87K
RMSD P88L
RMSD E92D
RMSD R97C
RMSD H111R
RMSD S115F
RMSD S118F
RMSD R131G
RMSD I145M
RMSD A153T
RMSD R161W
RMSD G202C
RMSD R206C
RMSD Q226H
RMSD R229Q
RMSD Q263H
RMSD V267M
RMSD T285M
RMSD P291Q
RMSD S292N

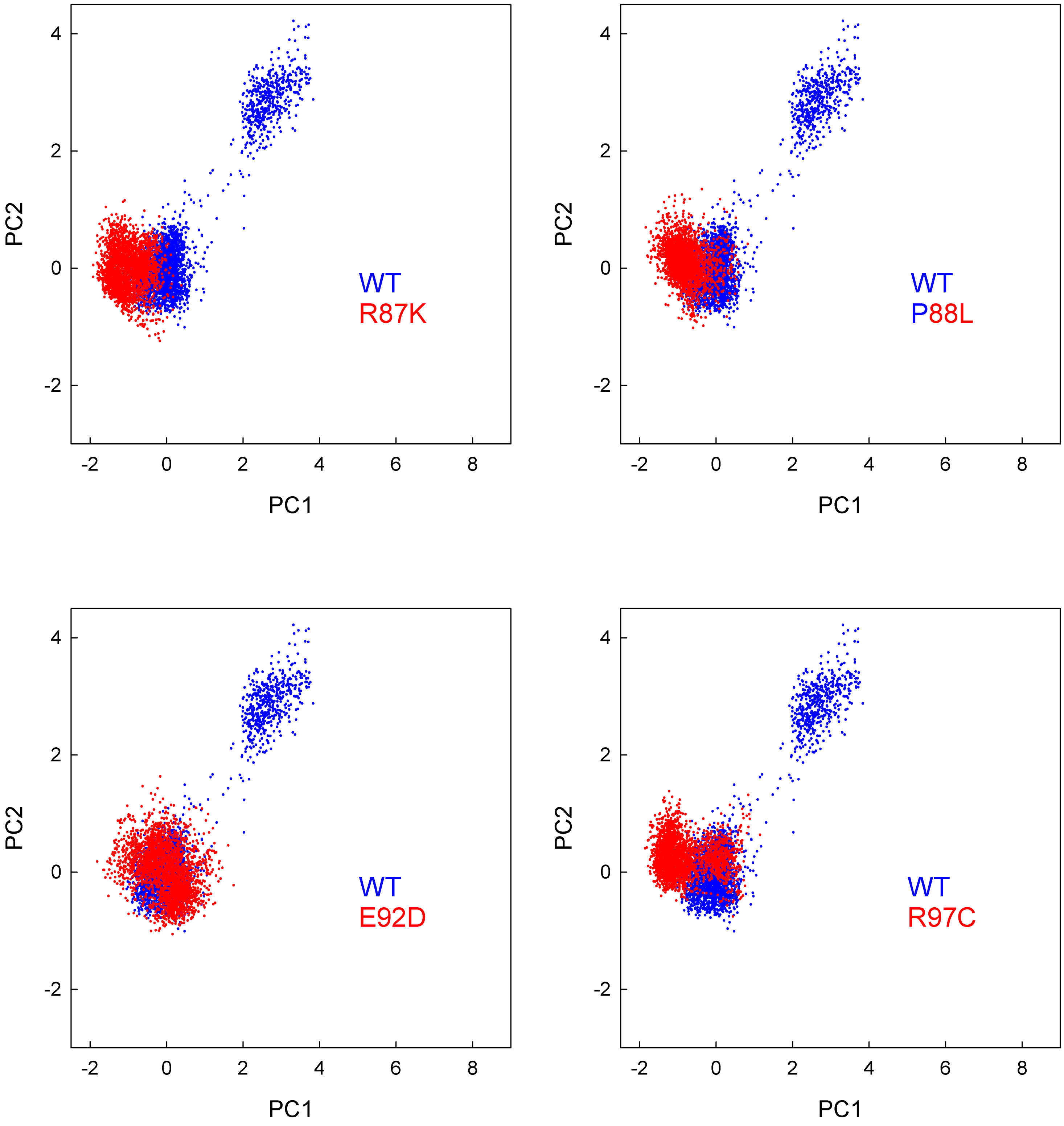

Supporting Figure S2 (part 1 of 5)

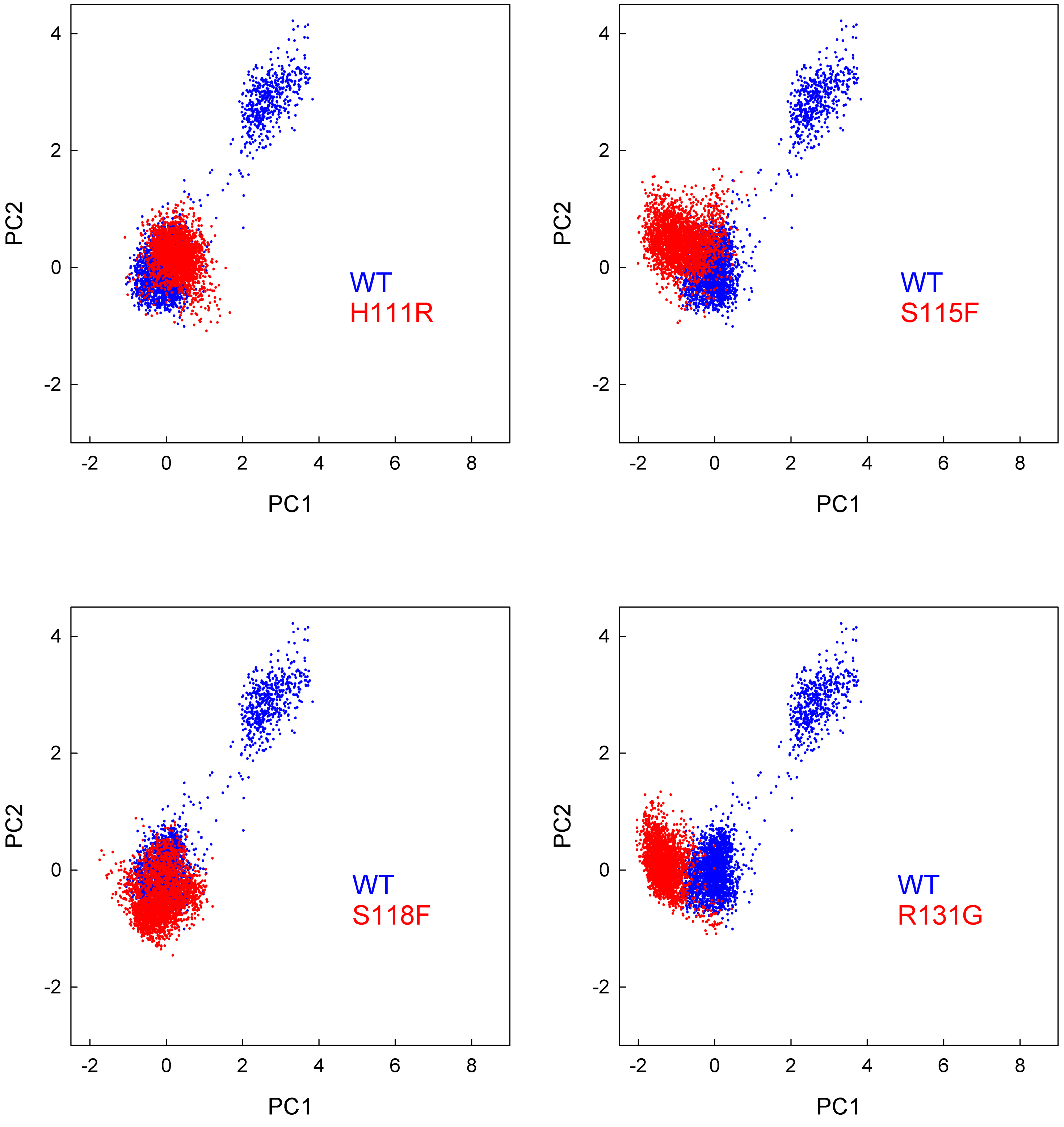

Supporting Figure S2 (part 2 of 5)

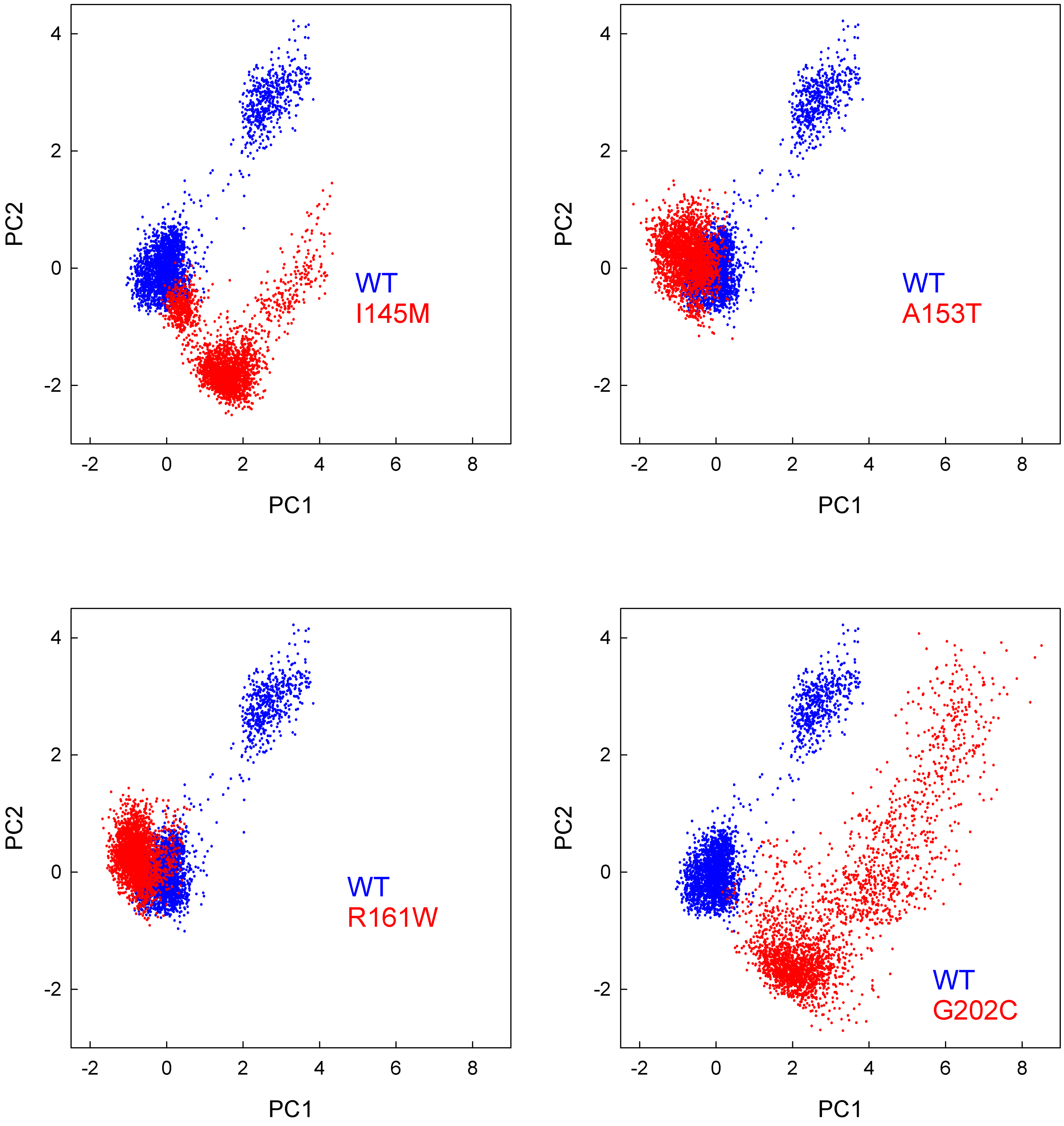

Supporting Figure S2 (part 3 of 5)

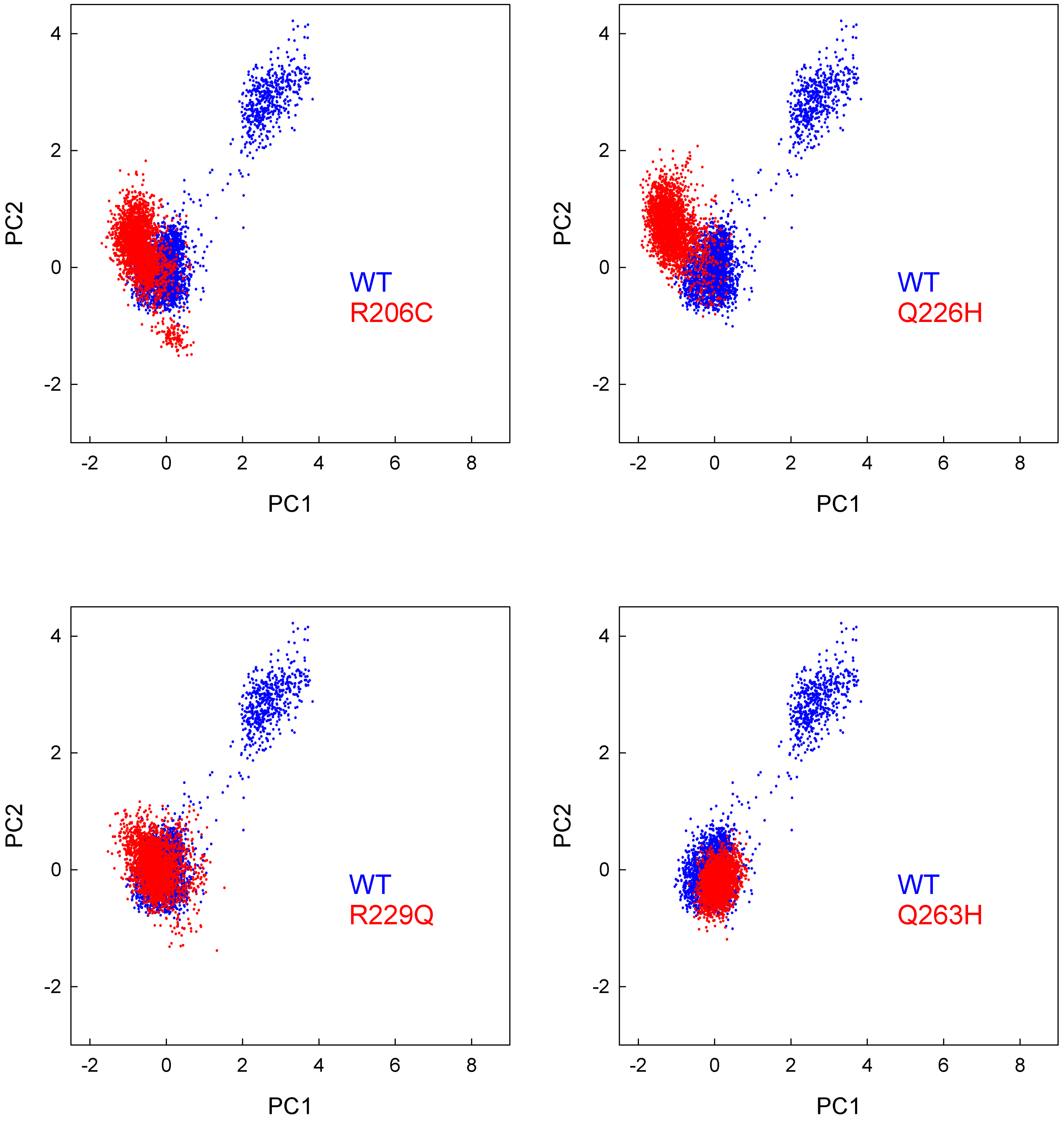

Supporting Figure S2 (part 4 of 5)

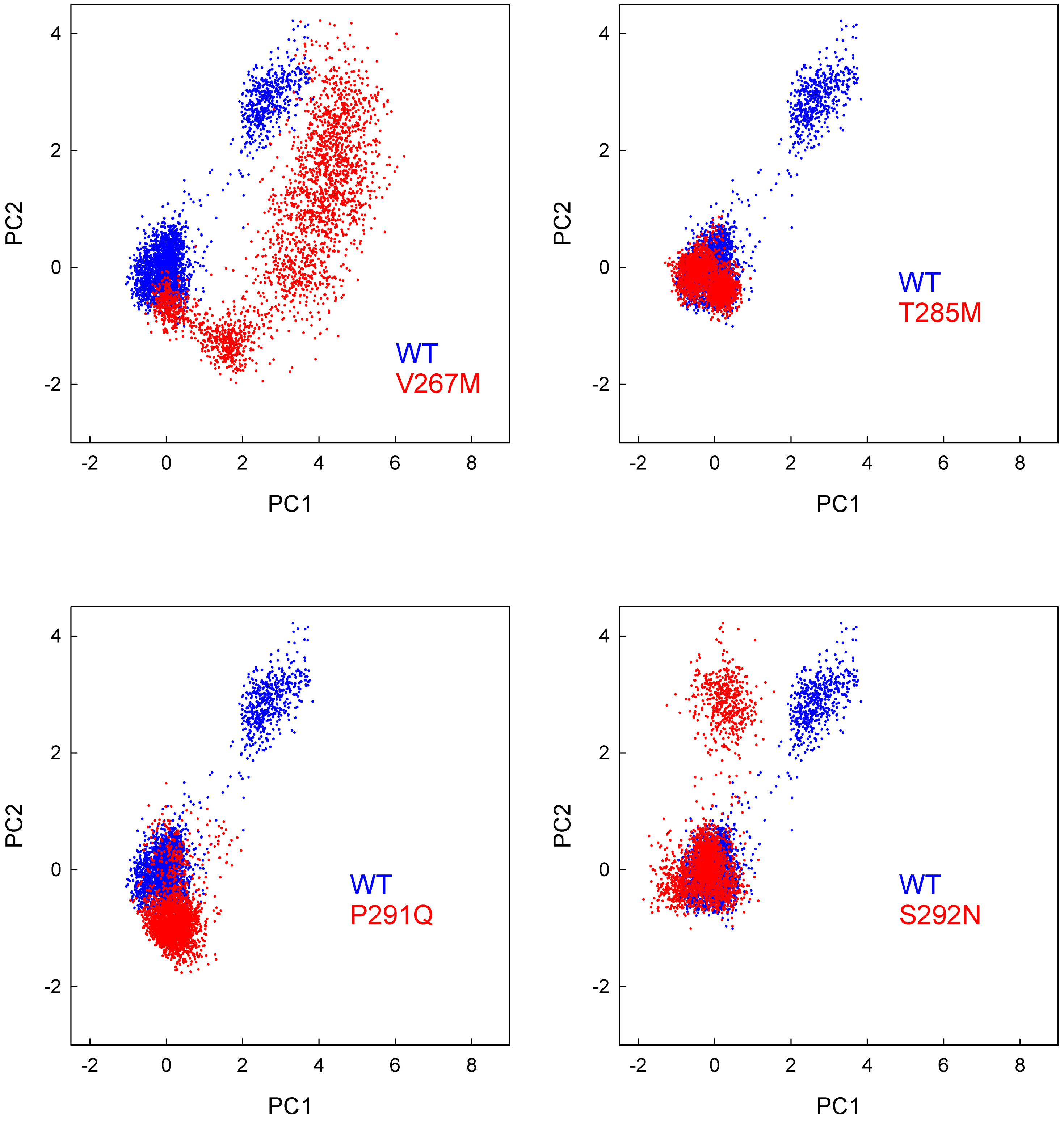

Supporting Figure S2 (part 5 of 5)

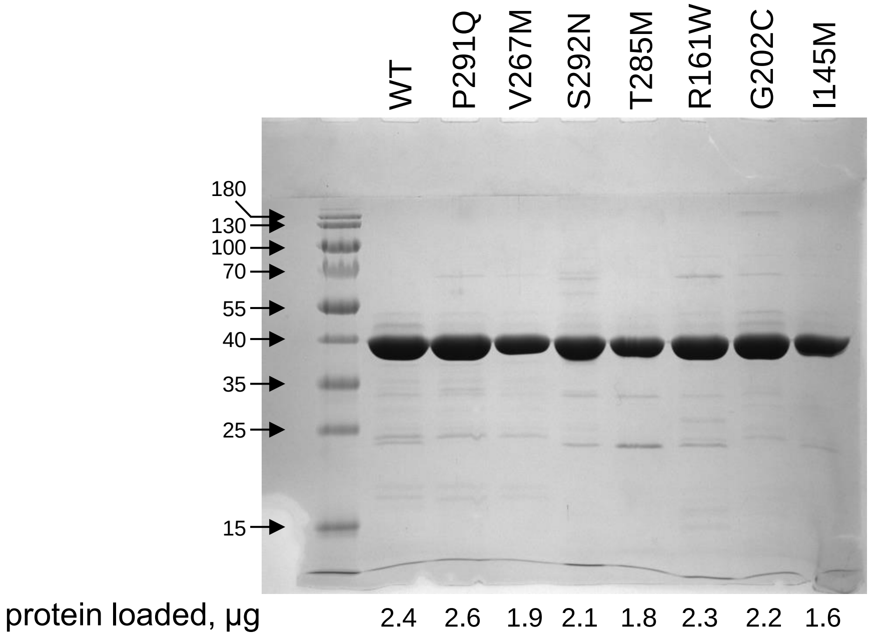

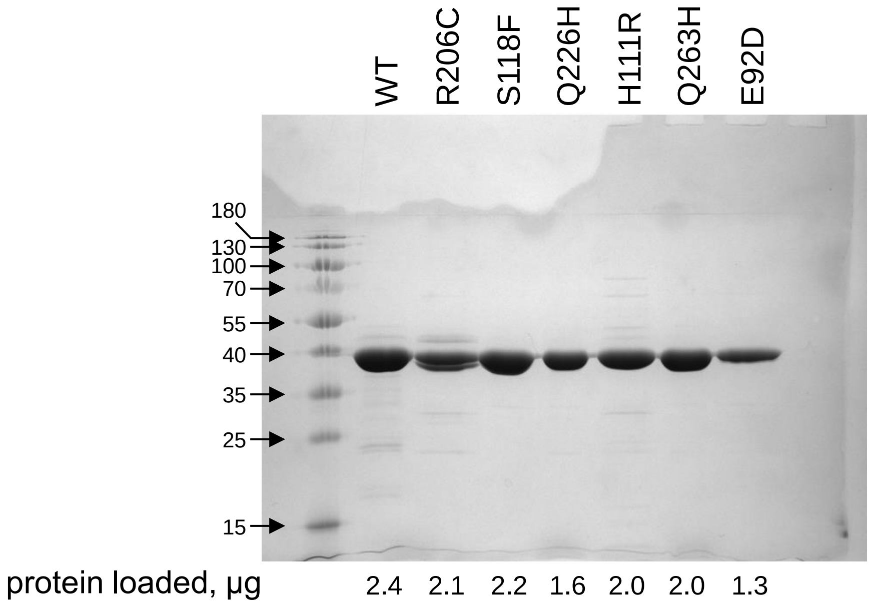

Supporting Figure S3

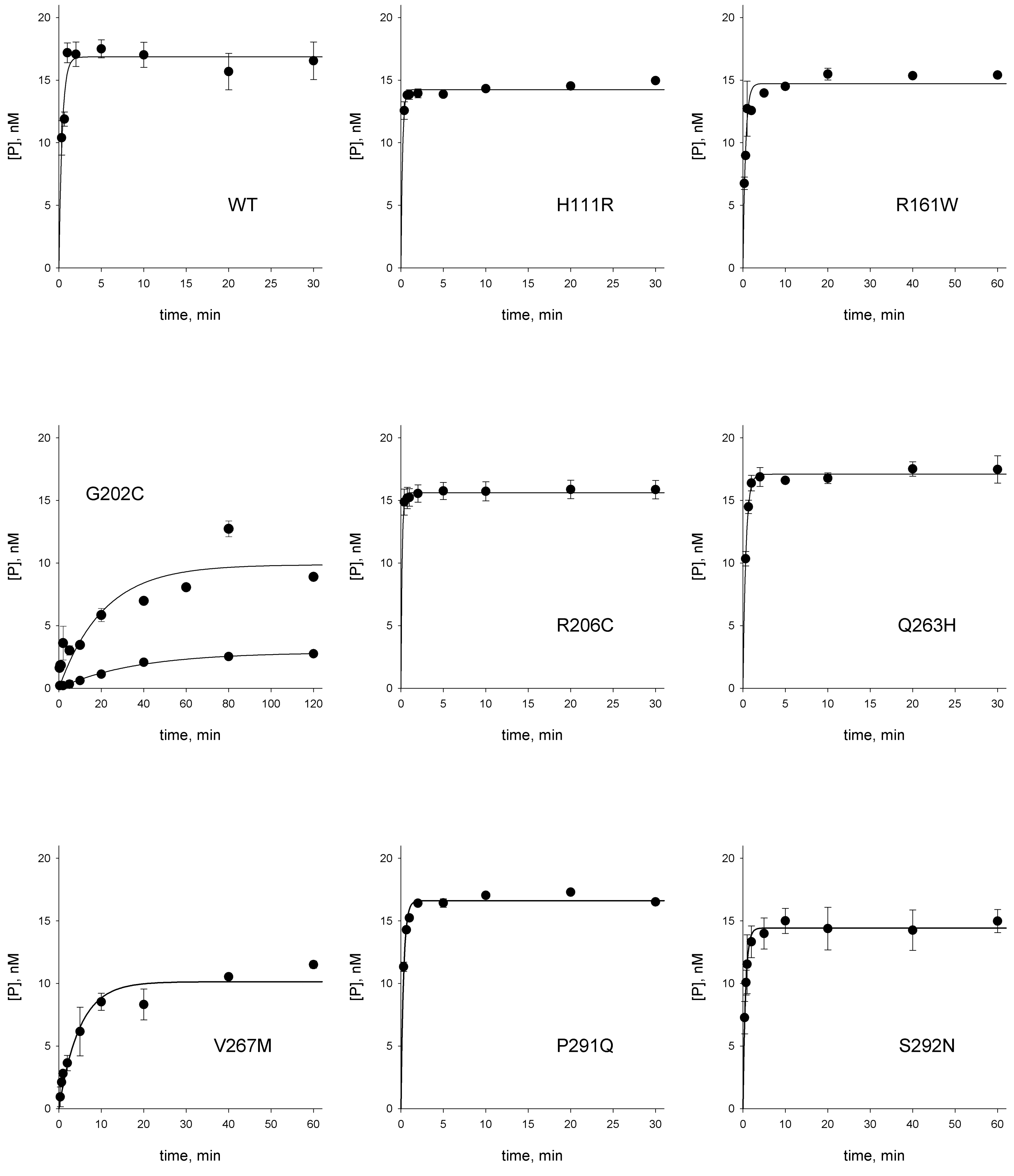

Supporting Figure S4

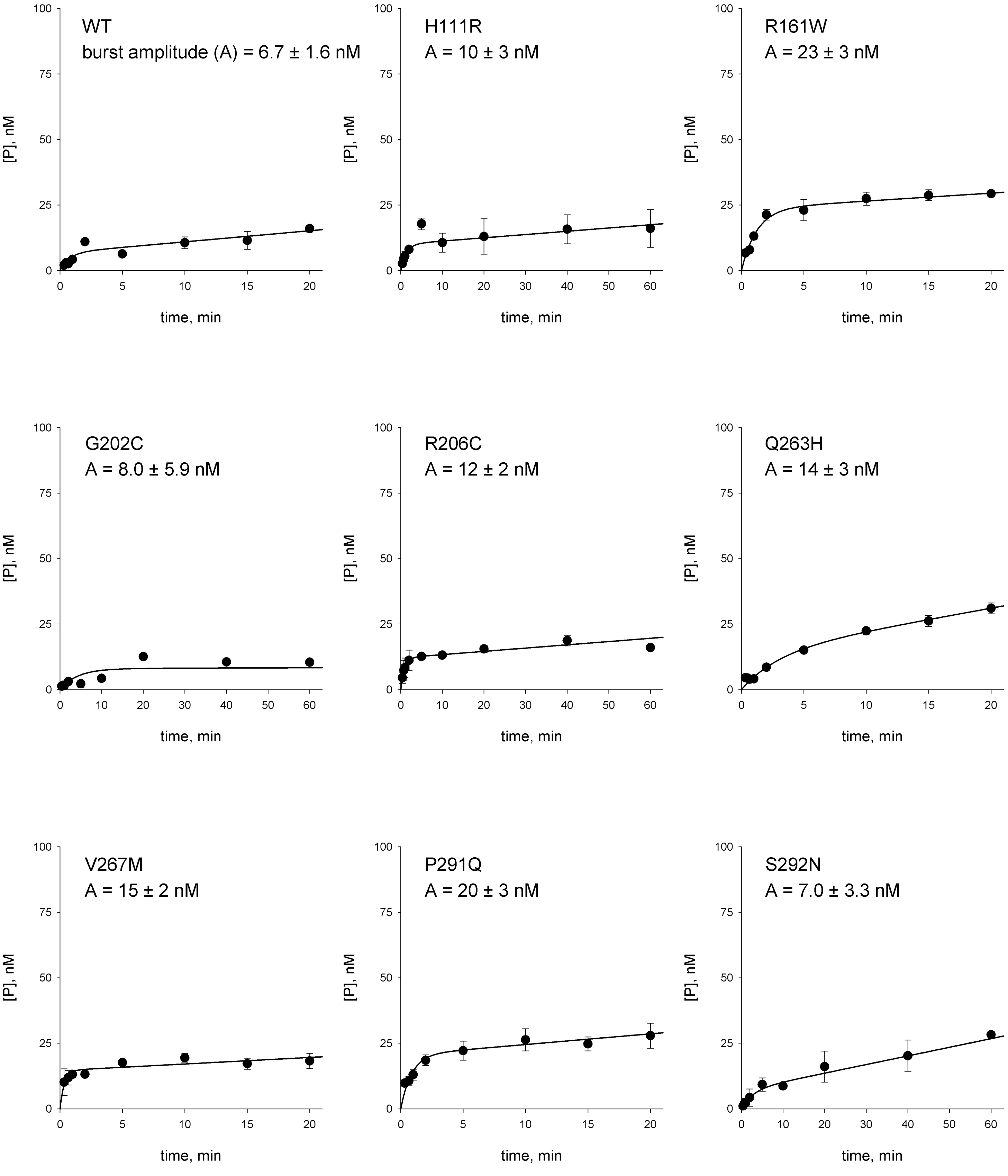

Supporting Figure S5

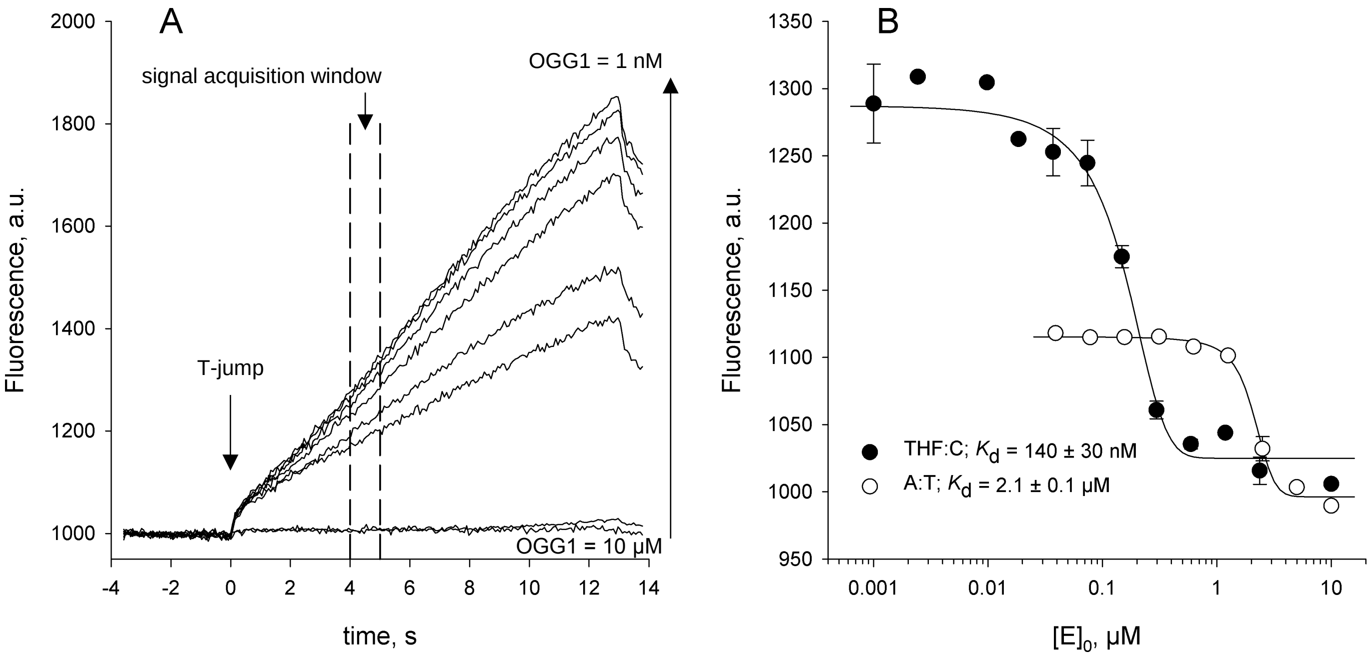

Supporting Figure S6
